# Characterization of PLA Sheets Prepared by Stretching under Different Conditions: Influence of Reprocessing and Establishing Optimal Conditions

**DOI:** 10.3390/ma16145114

**Published:** 2023-07-20

**Authors:** Zaida Ortega, Paula Douglas, Paul R. Hanna, Graham Garrett, Alan Clarke, Eoin Cunningham, Luis Suárez

**Affiliations:** 1 Departamento de Ingeniería de Procesos, Universidad de Las Palmas de Gran Canaria, 35017 Las Palmas de Gran Canaria, Spain; 2 Polymer Processing Research Centre, School of Mechanical and Aerospace Engineering, Ashby Building, Queen’s University of Belfast, Belfast BT9 5AH, Northern Ireland, UK; p.douglas@qub.ac.uk (P.D.); p.r.hanna@qub.ac.uk (P.R.H.); g.s.garrett@qub.ac.uk (G.G.); ah.clarke@qub.ac.uk (A.C.); 3 School of Mechanical and Aerospace Engineering, Ashby Building, Queen’s University Belfast, Belfast BT9 5AH, Northern Ireland, UK; e.cunningham@qub.ac.uk; 4 Departamento de Ingeniería Mecánica, Universidad de Las Palmas de Gran Canaria, 35017 Las Palmas de Gran Canaria, Spain; luis.suarez@ulpgc.es

**Keywords:** PLA, biopolymer, biaxial stretching, processing conditions, characterization

## Abstract

Polylactide (PLA) is one of the most commonly used biomaterials nowadays, with many recognized benefits, particularly in the packaging and single-use products industries. However, little research has been conducted on its stretching behavior. This work investigates the optimal conditions of biaxial stretching of injection-molded PLA samples produced under different processing conditions (pressure, drying, and pre-processing by extrusion, to simulate a recycling step). The injection-molded samples were characterized to determine their mechanical, thermal and thermo-mechanical behavior, water absorption, thermal behavior, and crystallization kinetics. The extruded samples showed reduced thermal stability, lower viscosity, decreased mechanical properties, and higher crystallization rates due to thermal degradation. However, the stretched samples provided similar properties regardless of the materials pre-processing. Regarding the assessment of the biaxial stretching process, processing at lower temperatures provides the films with a higher yield and breaking strength, while the time and strain rates have little influence on such properties. It was then determined that 82 °C is the optimal temperature for stretching the PLA samples. An increase in the stretch ratio provided a higher elastic modulus and higher values of opacity due to an increased crystallinity induced by stress during the process. Films as thin as 50 μm can be obtained by biaxially stretching injection-molded preforms, producing a deformation over 150% and acquiring good mechanical properties: about 90 MPa for the yield and a breaking strength and elastic modulus of 4000 MPa.

## 1. Introduction

The use of disposable plastics in daily life has become one of the most critical environmental problems due to the high stability of these materials when not disposed of adequately, the consumption of resources needed for their production, the massive quantity of residues, or their low recycling rates. In this sense, there has been some movement toward the reduction of single-use plastics, as outlined in Directive 2019/904 of the European Parliament and of the Council, which aims at reducing the impact of plastics on the environment. This regulation bans the use of single-use plastic in products commonly found in the environment, including straws, cutlery, or packaging [1], thus encouraging a shift towards more sustainable alternatives. One solution is to use compostable or biodegradable plastics, such as polylactic acid (PLA) [2].

PLA is a biodegradable polymer under certain conditions of temperature and humidity [3], which can be obtained from petrochemical sources as well as from biomass through the fermentation of sugars-rich substrates, such as sugar cane, wheat, or rice [4]. In 2021, the global PLA market was valued at over USD 550 million, and it is estimated that its production and demand will continue increasing by over 25% until 2030. Packaging is currently one of the sectors with higher use of this material, accounting for over a third of the total [5]. In fact, the packaging industry is the highest consumer of plastics globally [6,7].

While PLA can be used in injection and thermoforming processes to obtain several products, such as packaging items, its use for flexible applications requires blending with softer polymers [2]. PLA has properties similar to polyethylene terephthalate (PET) or polystyrene (PS), such as high rigidity, glossiness, and transparency, so it can replace them in several applications. PET has traditionally been one of the most used materials in the packaging industry, especially for the fabrication of bottles and films [8], due to its transparency and good barrier and mechanical properties. Single-use products made of PLA have a lower environmental footprint compared to PET or polypropylene (PP) [9], while providing similar mechanical properties. The main drawback of PLA is its lower thermal stability due to its lower softening temperature of about 60 °C [2,6].

The narrow processing window of PLA and its high sensitivity to chain scission by thermal and hydrolytic degradation make the recycling of PLA products complex [9,10], and more efforts are needed to assess and maximize the recyclability of PLA products. Despite this being a more favorable option for environmental behavior than biodegradation, bioplastics cannot yet be recycled in conventional processing plants and are mainly sent to landfills. These materials are easily affected by moisture and thermal cycles, resulting in the deterioration of their properties. Some authors have proposed using chain extenders or incorporating other polymers, such as PBAT or PCL [4,11], to increase the polymer toughness and reduce its brittleness, potentially widening the range of applications and improving its thermal stability.

The degradation of PLA results in lowered mechanical properties, a yellowish color, and changes in the crystallization behavior and rheology due to the changes in the molar mass [12]. The grade of PLA used plays a role in the degradation behavior of the polymer; those with a higher fluidity (lower viscosity) are less likely to degrade [12]. The D-isomer content and crystallinity also influence the degradation and reprocessing possibilities; the higher the D-isomer content and the lower the crystallinity, the greater the degree of degradation [13].

Processing conditions, including how the material is dried, affect PLA behavior. For example, Signori et al. found that molecular weight decreases with an increasing processing temperature and humidity content: if the material is not dried, the reduction in the molecular weight is higher than if it is dried. Drying under a nitrogen atmosphere instead of air slightly reduces this drop, but only for higher processing temperatures (over 200 °C) [14]. Speranza and collaborators [15] arrived at similar conclusions when studying the rheology of undried and dried PLA: the presence of humidity affects, to a great extent, the viscosity due to hydrolytic degradation of the polymer during processing. Zenkiewicz et al. [16] also found that subsequent processing of PLA results in reduced performance, as evidenced by decreased viscosity (measured as Melt Flow Index, MFI) and increased crystallinity.

Two of the most commonly used manufacturing processes within the packaging industry are injection-stretch blow molding and thermoforming. The behavior of polymer materials used in these processes can be simulated under biaxial stretching. This technique allows for establishing the properties and processability of the materials [17], which are determinant for the process economy and environmental performance. The idea behind this procedure is to perform a semi-solid deformation on injection-molded preforms under different parameter processes: temperature, heating time, elongation rate, and stretch ratio. Several studies, mainly conducted on PET, have established that the mechanical properties of the material after processing depend on the strain rate, the deformation mode, and the stretch ratio [18]. Some works have already shown similar properties on vacuum-formed parts and biaxially stretched films, demonstrating the applicability of this technique [19]. PET is able to crystallize under strain (strain-induced crystallization: SIC), which explains the high thermomechanical stability of blown-molded PET products [20,21]. Several authors have shown that SIC occurs only in the rubbery state, at temperatures slightly over glass transition (T_g_), and does not occur under low strain rates due to molecular relaxation. 

Blair et al. performed some research on the study of biaxial stretching of PLA films to produce coronary stents [22,23]. These authors found that SIC can also take place in PLA when working over T_g_ without reaching a cold crystallization temperature (T_cc_), as was also explained by Ou and Cakmak [24]. These latter authors also pointed out that cold crystallization reduces entropy, results in strain hardening, and allows self-leveling, thus leading to films with uniform thickness. Blair and collaborators found that the processing history plays an essential role in the properties of the stretched parts [22,23]. Guinault et al. also studied this process on 100 μm thick extruded PLA sheets that were later subjected to an annealing process. These authors determined a maximum stretch ratio of 4, with no modifications in the barrier properties due to the stretching process and increases in crystallinity [25]. Tsai et al. investigated the stretching of PLA-extruded material to obtain thin films, and found that the optimal conditions for force-oriented crystallization are between T_g_ (about 60 °C) and 80 °C. A thermal analysis of biaxially stretched films shows a higher glass transition temperature than the extruded ones, suggesting the alignment of the polymer chains, which also led to obtaining a transparent film [8]. Yu et al. found that stretching increased the crystallinity, but also allowed for chain relaxation, increasing the elastic modulus and toughness of the PLA films [26]. Ou and Cakmak observed a decrease in the T_cc_ and a rise in the crystallinity in stretched PLA films, although they used a PLA with 8% of D-isomer, which is relatively high [24]: crystallization rises as deformation increases. Furthermore, annealing of the stretched samples enhances the crystalline order and leads to in-plane isotropy [27]. Some other authors have proposed the use of PLA-extruded films to produce coronary stents by blow molding [22,23,28,29,30,31]. In such works, the extruded PLA films (96 % L-isomer) are stretched in two directions, and the behavior of that PLA grade is simulated under a glass–rubber model. Different parameters were used in these works, with temperatures ranging from 80 to 100 °C, strain rates from 1 to 16 (1, 4, 16 s^−1^), and stretch ratios from 1 to 3. Similar research was conducted by Wu et al. [32], with a stretch ratio up to 4, temperatures between 90 and 110 °C, and a strain rate of 10–100%/s, under 1 min of heating (for the grade of PLA used in this work, the degree of L or D-isomers is unknown).

However, these works were mainly focused on obtaining films and are not particularly aligned with blow molding or thermoforming, as they usually start from an extruded film rather than injection-molded samples. A recent study by Licea-Saucedo et al. [33] included an analysis of blow-molding of different PLA grades, and found that temperature influences, to a great extent, the mechanical properties of the obtained films; instabilities were also encountered during the process. They found a high dependency between the temperature and the processability; the tensile strength of the films was independent of the stretching and blow-up ratios for PLA LX175, with an average value of 49 MPa. These authors determined that the molecular weight of the PLA plays a determinant role in the processability; a lower molecular weight (thus, lower viscosity) produces inconsistent films with a high variability of properties.

In contrast with the research that has been performed to-date, this paper focuses on the study of biaxial stretching of various injection-molded PLA samples (Luminy L105) due to the sensitivity of this material to processing conditions. The effect of the processing conditions (drying cycle, extrusion, and injection molding parameters) on the properties of the material (DSC, crystallinity, kinetics of crystallization, behavior under biaxial stretching, and tensile behavior of stretched sheets) has been determined as a method to determine the suitability of such material for thermoforming or blow molding. A Doehlert design of the experiments with three levels was performed to determine the optimal conditions of the stretching process: temperature, time, and strain rate, fixing the stretch ratio parameter to 3. The temperature limits were determined according to the results obtained from the thermal characterization of the materials processed via the different methods and on the basis of the preliminary tests conducted.

The optimization of such parameters was carried out through the equations obtained by the response surface methodology (RSM), finding the optimal values for temperature, time, and strain rate. To the best of the authors’ knowledge, this is the first study where the optimization of the parameters affecting the stretching of polymer (PLA) parts is performed under RSM. The use of such methodology, together with a design of experiments, allows for reducing the number of samples used while still obtaining consistent results. Once these parameters were defined, the stretch ratio was increased, and the samples obtained were further characterized.

The grade of PLA used shows a very low D-isomer (lower than 1%, according to the manufacturer), which is expected to provide a high rate of crystallization. This research constitutes a previous step to the assessment of the composite materials containing naturally derived fillers, which could help reduce the amount of material needed to reach some specific properties (i.e., yield strength), which has a significant impact on the economy and environmental footprint of the process.

## 2. Materials and Methods

### 2.1. Material Preparation: Drying, Extrusion, and Injection Molding

Biaxial test specimens of dimensions 76 × 76 × 1.2 mm were obtained by injection molding of PLA Luminy L105 from Corbion Polymers. The injection molding process was performed using an Arburg 320S injection machine, with the following temperature profile from back to nozzle: 190–195–200–205–210 °C with a back pressure of 100 bar, a flow rate of 70 cm^3^/s, and a holding pressure of 250 bar. The switchover pressure was recorded for the different batches. The mold temperature was kept at 30 °C.

The material was prepared in different ways in order to determine how the processing (drying time, extrusion, and injection molding) influences the behavior of the material. Table 1 shows the variations performed and the short names for each processing condition (injection molding was conducted using the same conditions for all the samples, except for the so-called optimal parts, as explained below). The samples processed from virgin pellets and short-dried (named “L105” and “SD”, respectively) followed the same drying process, with the difference between them being that L105 was used as purchased, while SD was further processed by extrusion. The samples processed through a long drying step (named “LD”) were dried before extrusion, as in the SD, but with a longer drying time after the extrusion process. In short, the SD and LD were extruded in the same conditions, with the only difference between them being the drying time after extrusion (before the injection molding). A final set of samples, dried and processed under the same conditions as the injected L105, at the minimum injection-molding flow rate possible, was obtained, as recommended in the polymer datasheet, and named “Opt” (from optimal conditions, as these samples also provided a more transparent aspect, without any fogging). For this set, the volumetric flow rate was reduced to 50 cm^3^/s, and the holding pressure was increased to 350 bar.

The extrusion of the material was performed in a Collin ZK25 extruder, with a 25 mm diameter, at a screw rotation speed of 250 rpm and a temperature of 190 °C. The aim of processing the virgin material in this way was to simulate a mechanical recycling step. In a similar way, the comparison between the two drying times aims to determine if this longer step results in a modification of the properties of the material. PLA is well-known to degrade due to thermal processing and hydrolysis, if humidity is involved. It is therefore considered of interest to determine wether the drying step influences the properties of the final product. The different materials were dried for 4 h at 100 °C before injection molding.

### 2.2. Materials Characterization

The Fourier transform infrared (FTIR) spectra were obtained in a Spectrum 100 spectrophotometer from Perkin Elmer under the attenuated total reflectance (ATR) mode. A total of 64 scans were recorded per spectra, at a resolution of 4 cm^−1^, in the range of 4000 to 600 cm^−1^.

The melt flow index (MFI) was determined at 190 °C, with a load of 2.16 kg, according to ISO 1133 [34], using a 7053 device from Kayeness Inc (Dynisco Company, Franklin, MA, USA). The results are given as the average of 5 samples. 

Water absorption was carried out according to ISO 62:2008 [35]. The specimens were immersed in deionized water and weighed periodically until the weight was constant. The water absorption was calculated using the following equation:

(1) $$W\left(\%\right)=\frac{W_{t}-W_{0}}{W_{0}}\cdot 100$$

where *W*_0_ is the initial weight of the sample, *W_t_* is the weight of the sample at time *t*, and *W*(%) is the percentage increase in weight. Three replicas per sample were assessed.

The kinetics of water uptake can be obtained using Fick’s law:

(2) $$D=\pi \cdot {\left(\frac{k\cdot h}{4\cdot W_{m}}\right)}^{2}$$

where *D* is the diffusion coefficient (m^2^/s), h is the thickness of the original sample, *W_m_* is the maximum moisture absorbed by the sample, and *k* is the initial slope of the curve of water uptake versus *t*^1/2^, as described by Equation (3) [36]:

(3) $$k=\frac{W_{2}-W_{1}}{\sqrt{t_{2}}-\sqrt{t_{1}}}$$

If the moisture uptake at each measurement is compared with the maximum water uptake of each sample, the parameters *n*, associated with the diffusion mode, and *k*, related to the interaction between the material and the water, can be calculated as follows [37]:

(4) $$\frac{W_{t}}{W_{m}}=k\cdot t^{n}$$

Differential scanning calorimetry (DSC) was performed on all the samples in a Perkin Elmer DSC 6 apparatus under a nitrogen atmosphere. Samples of approximately 5 mg were prepared in closed aluminum crucibles. The measurements were performed at 10 °C/min, from 30 to 200 °C, with two heating cycles. The melting temperature for both the heating cycles (T_m1_ and T_m2_, respectively) and the crystallization temperature (T_c_) from the cooling step were determined, together with the melting and crystallization enthalpies (ΔHm_1_, ΔHm_2_, and ΔHc). The enthalpies were used for the crystallinity degree (χ) calculation using the following expression:

(5) $$\chi =\frac{\Delta H_{m}}{\Delta H_{0}}\cdot 100$$

where Δ*H*_0_ is the enthalpy for the 100% crystalline sample (93.7 J/g) [10]. Three tests were performed per material type, and the results are given as average values (less than 5% deviation for all the series).

The isothermal crystallization kinetics were determined using the same apparatus. Samples of approximately 5 mg were prepared in sealed aluminum crucibles under a nitrogen atmosphere and subjected to a first stage of heating from 30 to 200 °C at 30 °C/min, and then kept at 200 °C for 5 min to remove the thermal history of the material. Then, the sample was cooled down to the desired temperature at the same rate and kept at the temperature of the study for 30 min. Finally, the sample was heated again until it reached 200 °C for 5 °C/min to determine the values of crystallinity from the melting enthalpy.

The flow properties of the materials were assessed in an oscillatory rheometer AR G2, from TA instruments, with 25 mm diameter parallel plates and a 1.5 mm gap under a nitrogen atmosphere. The experiments were conducted at 210 °C. Preliminary assays were performed under the strain sweep mode in order to ensure that the later experiments were placed in the linear viscoelastic region (LVE). In these tests, the strain was varied between 0.1 and 5%. Frequency sweep tests were performed at 0.5% strain, in the LVE, in the 100 to 0.01 Hz range. Finally, flow tests were also performed at the same temperature, between 0.01 and 1 Hz shear rate.

The tensile properties of the injection-molded samples were determined following ISO 527–2:2012 [38], at a rate of 1 mm/min for the ultimate tensile strength and 0.25 mm/min for elastic modulus. The flexural properties were measured according to ISO 178:2019 [39] at 1 mm/min, with 22 mm between the cantilevers, determining the elastic modulus and flexural strength. The tensile and flexural tests were performed using an LS5 universal testing machine from Lloyd, with a cell load of 500 N for the modulus and 5 kN for strength, with 5 replicates per test. Charpy impact tests were performed on notched samples, following UNE-EN ISO 180:2019 [40], using a 7.5 J pendulum and an impact rate of 3.7 m/s in a Ceast Resil impactor P/N 6958.000, with 10 replicates. The results are given as average values and standard deviation (SD).

The thermomechanical properties of the different materials were evaluated by dynamic mechanical thermal analysis (DMTA) using a Tritec 2000 device, from Triton Technology, under the single cantilever bending method. A strain of 10 µm was applied at 1 Hz frequency between −50 and 120 °C, with a heating rate of 2 °C/min.

The color of the samples was assessed following the International Commission on Illumination (CIE) L* a* b* coordinates. In this system, L* is the color lightness, whereas a* and b* represent the coordinates of redness (green (−)/red (+)) and yellowness (blue (−)/yellow (+)). These parameters were determined by optical spectroscopy using an X-Rite SP64 portable spectrophotometer, measuring 5 samples per material. The total color difference parameter (ΔE*) was calculated as indicated in Equation (6) following ISO 7724 [41] standard:

(6) $$\Delta E^{*}={\left[{\left(\Delta L^{*}\right)}^{2}+{\left(\Delta a^{*}\right)}^{2}+{\left(\Delta b^{*}\right)}^{2}\right]}^{0.5}$$

The opacity was calculated using the same device by measuring the sample against a white and a black background. Finally, the yellowness index (*YI*) was used to quantify the degradation of the PLA due to its processing and was calculated as follows:

(7) $$YI=\frac{142.86\cdot b^{*}}{L^{*}}$$

### 2.3. Biaxial Stretching

#### 2.3.1. Biaxial Stretching Experiments

**Figure 1:**
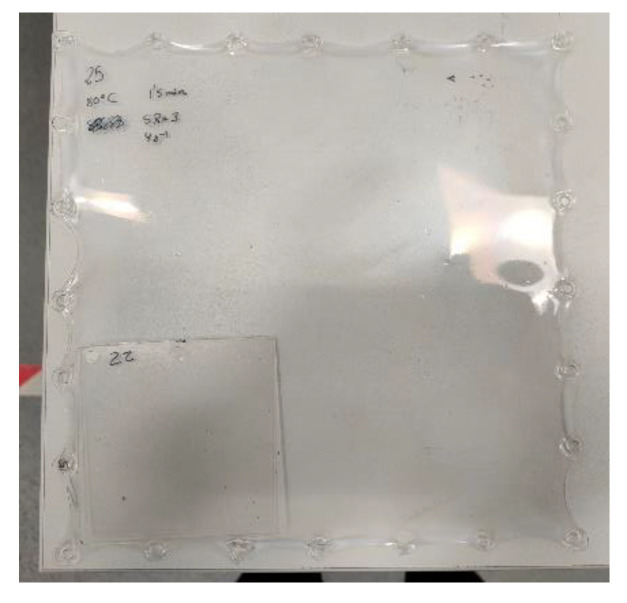
Injection-molded 76 mm square test plaque and biaxially stretched sheet at a stretch ratio of 3.

Biaxial stretching was performed using the Queen’s biaxial stretcher (QBS), which was developed at Queen’s University of Belfast, to simulate the behavior of polymers in thermoforming and blow molding [17]. The stretching parameters were defined following preliminary testing trials and included a range of temperatures, heating times, stretch ratios, and strain rates. The tests were performed under equal biaxial stretching mode, that is, stretching the sample in the x and y direction at the same time. Each test was repeated twice while recording the force and displacement in the x and y-axis. The results are given as the average value of the performed tests and are provided as the true stress and strain. Figure 1 shows the comparison in size between an injection-molded test plaque and a biaxially stretched sheet with a stretch ratio (SR) of 3.

#### 2.3.2. Optimization of Biaxial Stretching Parameters

In the first step and according to preliminary tests and literature to define the boundaries of each parameter, the stretch ratio (SR) was fixed to 3. The influence of the remaining three factors was evaluated at three levels, following a Doelhert design: temperature (from 80 to 95 °C), strain rate (from 1 to 32 s^−1^, equivalent to stretch speeds of 33.5 to 1072 mm/s), and heating time (from 45 to 90 s). These experiments allowed the optimal values for such parameters to be defined and used in a later stage of stretch ratio modification. The conditions used are summarized in Table 2.

**Figure 2:**
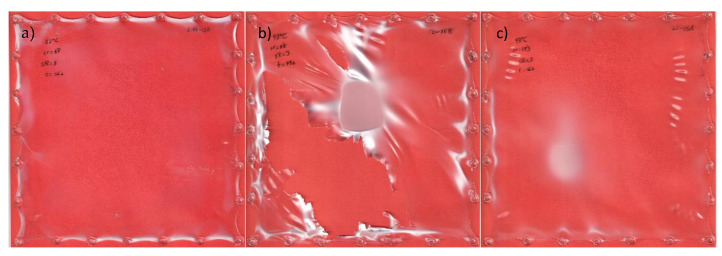
Pictures of samples after biaxial stretching showing: (**a**) good processability, (**b**) bad processability (breaking), and (**c**) good stretching but whitening.

The response variables of the experimental design were the processability of samples under the conditions used and the forces measured in both directions. As discussed in the results Section 3.10, a value of 1 was assigned to samples that stretched without breaking and without whitening, 0 for those samples that broke during the process, and 0.5 for those that were correctly stretched but showed whitening, thus leading to poor aesthetics (Figure 2) and higher variability of the results.

For each response, the average values obtained from the duplicates were fitted to a second-order polynomial model, as shown in Equation (8), with A and B representing the parameters combined. In order to obtain the plots and optimize the parameters, an analysis was made by a pair of variables, allowing us to obtain the parameters of the equation for the relationship between the time and temperature, the time and strain rate, and the temperature and strain rate.

f(A,B) = p00 + p10 × A + p01 × %B + p20 × A^2^ + p11 × A × B + p02 × %B^2^ (8)

The response surfaces were plotted using Matlab, R2022b (Update 4) and the fit of the models to the experimental data was given by the coefficient of determination (R^2^). The optimum conditions were then numerically determined using the equations obtained for the fitting of experimental data.

Under the optimal conditions determined in this first step (temperature, time, and strain rate) for each series of samples, the influence of the stretch ratio was also assessed. Independent samples were subjected to stretch ratios of 3 and 4 (67.0 to 100.5 mm of deformation) to determine the processability and forces afterward in both directions, as well as the mechanical properties of the obtained sheets.

#### 2.3.3. Characterization of Stretched Sheets

The Obtained sheets were die-cut to develop test bars for the determination of the tensile properties, according to ISO 527–3:2012 [42], at a rate of 10 mm/min for the ultimate and breaking tensile strength and 1 mm/min for the elastic modulus. The results are given as average values and standard deviation (SD).

A dynamic mechanical analysis (DMA) was conducted on the biaxially stretched sheets in a Tritec 2000 device, from Triton technology, in the tensile mode in order to evaluate the T_g_ and storage and loss modulus (E′ and E″); the tests were performed from −50 to 120 °C, at a heating rate of 2 °C/min, a strain of 10 µm, and 1 Hz frequency.

The color measurement and opacity were measured as explained above, with 5 measurements obtained for each sheet.

## 3. Results and Discussion

### 3.1. Injection Molding

The processing of the virgin PLA affects its flow behavior to a great extent, as was found in the determination of the melt flow index. For virgin material, the MFI obtained was 33.8 ± 1.5 g/10 min, in line with the product datasheet, and it increased up to 75.5 ± 1.2 and 176.9 ± 5.2 g/10 min for SD and LD, respectively. That is, the extrusion of the polymer significantly increases its fluidity; thermal degradation seems to play a more critical role, as observed in the very large rise in MFI due only to the longer drying process. The drying process did not significantly affect the values of MFI, with values around 40 g/10 min obtained for the dried pellets with the longer time before their extrusion. However, it seems that the longer time at a high temperature followed by the extrusion process results in a significant modification (degradation) of the polymer.

These data correlate well with the values for switch overpressure recorded during the injection molding process, which varied from about 599–633 bar for the unprocessed materials (L105 and Opt series) to 492–539 for the extruded ones (SD and LD samples). The reduced viscosity of the polymer is an indication of its degradation; to minimize this effect, chain extenders, isocyanates, and other additives are being studied as a means to increase the recyclability of PLA products [9,33]. The start of the degradation can be observed in the yellowish color observed for the injection-molded samples, and is tabulated as yellow index in Section 3.9.

### 3.2. Chemical Analysis—Fourier Transformed Infrared Spectroscopy (FTIR)

**Figure 3:**
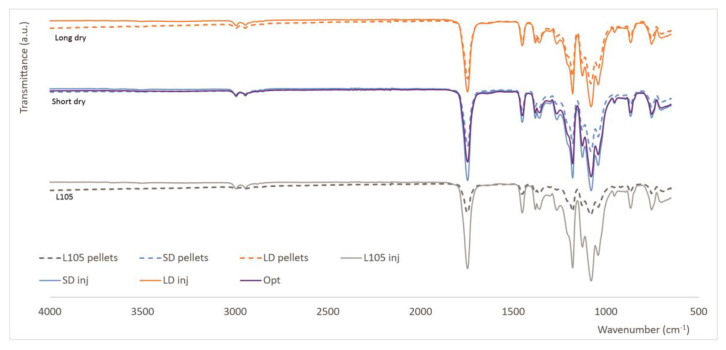
FTIR spectra for the pellets and injection-molded samples.

From Figure 3, which shows the FTIR spectra for the different processing conditions tested, it can be seen that there are no significant differences in the surface chemistry of the polymer due to its drying or processing by extrusion and injection molding; that is, no signs of degradation due to processing were observed in this analysis. The course of the curves show the typical fingerprint for PLA, that is, a carbonyl (-C=O) stretch in the lactide (1747 cm^−1^), a -CH_3_ bend (1465 cm^−1^, 1381 cm^−1^ and 1127 cm^−1^), and a C-O stretch (1180, 1078 and 1043 cm^−1^). The only difference was found between 2800 and 3000 cm^−1^, with a double peak for all the samples except for the virgin pellets; these bands are due to -CH stretching and are probably not visible in that spectrum due to the low values of transmittance found for the pellets (due to their shape). Different authors have proposed using the intensity of bands at 866 and 756 cm^−1^ as indicators of crystallinity, as these bands are attributed to the amorphous and crystalline phases of PLA [9,43]. Other works use the intensity of the band at 920 cm^−1^ as an indicator of crystallinity [13].

As it is well known that PLA is sensitive to humidity and processing, the spectra were normalized using the band for -CH_3_ vibration (1465 cm^−1^), as proposed in several works studying the degradation of PLA [9,44]. Of particular relevance are the bands at 1747, 1207, 1183, and 1085 cm^−1^, related, respectively, to carbonyl stretch, COC stretch, and C-O stretch. The thermo-oxidative degradation of PLA results in the formation of anhydride, carboxyl, and carbonyl groups; therefore, an increase in the bands at these wavelengths would be indicative of such degradation. The absorbance ratios calculated as the intensity of the peak at each wavelength divided by the absorption at 1465 cm^−1^ were calculated and compared (Table 3), and no specific trend was observed, which indicates that the processing of the material does not lead to any substantial surface chemical modification; in other words, the processing seems not to produce any significant degradation of the polymer. The only difference was found in the ratio at 920 cm^−1^, indicative of crystallinity, which tends to increase for all the processed material: from 1.04 ± 0.02 for the virgin pellets to 1.10 ± 0.04 and 1.12 ± 0.03 for the extruded pellets with short and long drying, respectively. This ratio also increases for injection molded samples to about 1.20 for all the samples.

### 3.3. Water Uptake

**Figure 4:**
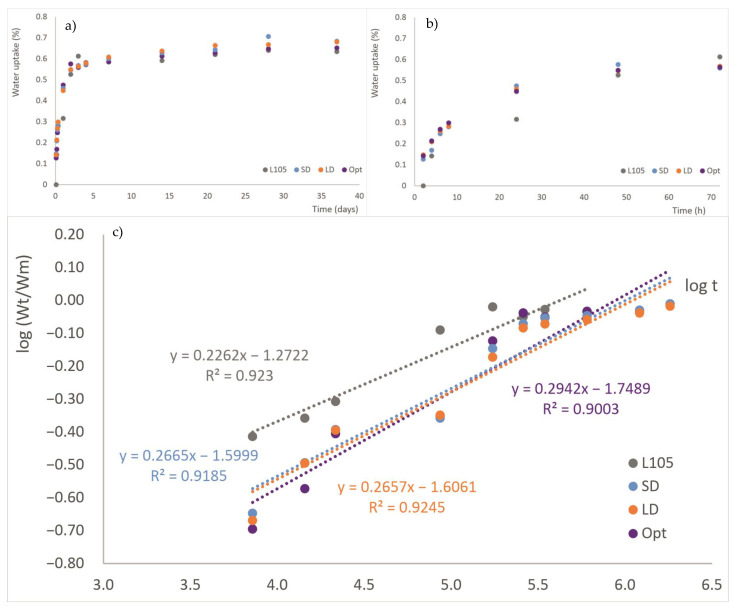
Results of moisture absorption. (**a**) Water uptake versus time, (**b**) water uptake versus time (in the first 3 days, in hours), and (**c**) calculation of Fick’s law parameters (log W_t_/W_m_ versus log time).

It is well known that water uptake is an essential step for the hydrolytic degradation of the material. From Figure 4a, it can be clearly observed that the processing does not affect the final water uptake of the sample, although if the first hours are analyzed (Figure 4b), it seems that the processing of the material increases the rate of water absorption, reaching about 0.5% for all the extruded samples and 0.3% for the injected virgin material after 24 h. Each point shown in the graph is the average value of three measurements. After 21 days of testing, where the maximum value for water uptake is found, a slight decrease was observed, which might be an indication of the beginning of PLA hydrolysis. The parts’ thicknesses were measured at the beginning and the end of the assay in order to determine their swelling, with values lower than 0.2% found in all cases, which means that the dimensional stability of the injected part is good.

From the data obtained, Fick’s diffusion coefficient can be obtained using equations 5 to 7. Figure 4c shows the representation of W_t_/W_m_ versus time in logarithmic scale, from which the parameter n is calculated. It can be seen that the data are well-adjusted to straight lines, with regression coefficients over 0.9 for all cases (Table 4). The parameter n indicates the type of diffusion behavior that is taking place and is found to be similar for all the series; that is, the different processing conditions do not affect the water diffusion mechanisms, as neither occurs for total the moisture uptake. This parameter is about 0.3 for all the samples, approaching Fick’s law (n = 0.5).

Some authors have calculated the sorption of water as the ratio between the mass of water absorbed by the sample and the mass of the test bar (Sorption, S), and then introduce the permeability (P) obtained by the combination of sorption and diffusion (P = D × S) [37]. It was observed that the SD and LD materials provided similar parameters. The lower diffusion coefficient and permeability were obtained for L105; the Opt parts demonstrated behavior closer to SD and LD than L105, which means that the injection molding conditions used for the production of this series (lower injection molding speed and higher holding pressure) affect the material in a similar way to the extrusion performed for those materials.

### 3.4. Thermal Behavior

The typical DSC curves for PLA were observed for all the samples, although with some significant differences among them, especially related to the appearance of the cold crystallization peak. The glass transition and melting temperatures occurred within the ranges indicated in the product datasheet, about 59 °C and 175 °C, respectively, as observed in Table 5. All the samples, except the virgin pellets, showed two exothermal peaks in both heating cycles: one at approximately 95 °C and a second, smaller one just before the melting peak (at approximately 160 °C). This last peak is related to the shift from α′ to α [8,45]. The α′-form is related to the disordered phase of PLA, while the α one is related to the ordered one, providing a lamellar morphology [46]. The material used has a low proportion of L-isomer, which tends to result in parts with relatively high crystallinity, although this is greatly dependent on the processing conditions; the manufacturer recommends performing the injection molding on a cold mold to obtain an amorphous structure and in a heated mold (100 °C) for a crystalline one. This temperature is the same that observed for cold crystallization in most of the samples (only some degrees lower for long-dried injection-molded samples). The process was performed at a mold temperature of 30 °C, obtaining transparent mostly amorphous parts, as shown in the DSC results (Table 5). The crystallinity of the unprocessed pellets was about 14%, while it was reduced to 8% for the extruded materials. This could be due to the rapid quenching in a water bath after the extrusion process. For the injection-molded samples, the crystallinity values were still relatively low, in the same range as for the pellets; again, this is due to the relatively rapid cycle of the injection molding process. The extruded materials provide higher levels of crystallinity than the virgin material, which might be related to the beginning of the degradation process during the extrusion process. It is also interesting to note that the material processed under the optimal conditions (lower injection rate, as recommended by the manufacturer, and higher pressure) shows a behavior closer to that of the virgin pellets. Finally, the solidification of the samples took place at about 95 °C, except for the virgin pellets, which is higher, and in the range of the crystallization temperature for the heating curve of the other samples.

**Figure 5:**
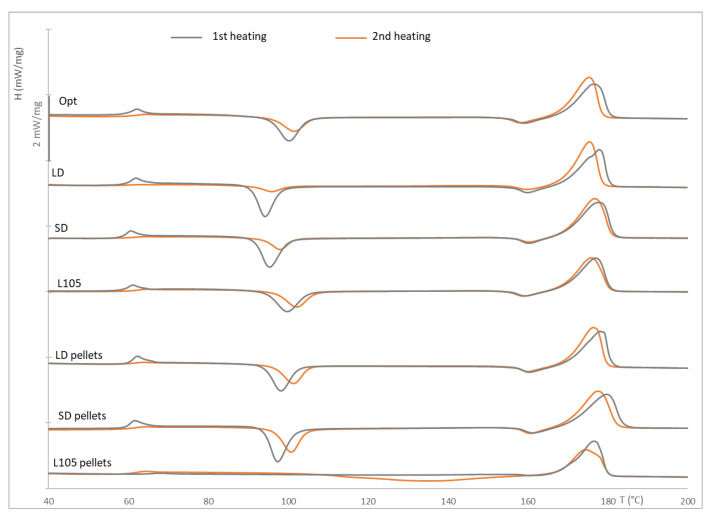
Thermogram for first and second heating of the different samples.

The differences are more clearly observed in the thermograms (Figure 5). The extrusion process clearly affects the thermal behavior of the material, transforming from a material unable to crystallize under the conditions of the assay (10 °C/min heating rate) to show a clear trend to crystallize. Tsai et al. [8] performed a similar study on extruded films and found no cold crystallization on the pellets without processing, but this appeared after the extrusion process. Stress suffered during processing helps in the molecular orientation of the molten polymer, thus providing the ability to crystallize.

On the other hand, the thermograms for the extruded materials are almost identical, regardless of the drying cycle. For the injection-molded samples, only the LD samples show a double melting peak, which might be explained by the ability of PLA thin crystals to melt and recrystallize at low heating rates, the existence of more than one crystal structure, or the presence of different lamella morphologies [47]. This was only seen in the first heating cycle, which also shows a more intense cold crystallization peak. Apart from this, and as already mentioned, the cold crystallization temperatures tend to decrease for processed materials, especially in the second cycle; this area is in the same range of temperatures for all the samples, while it is reduced (in temperature and area) for the injected long-dried material, which might be related to a higher ability to crystallize, potentially due to a higher extent of the materials’ degradation, as observed on the higher MFI values. The same trend was also observed in the values of crystallization enthalpies. Finally, the melting and glass transition temperatures of all the samples show no significant differences, which might suggest that there has not been a considerable modification of the polymer due to its processing, as was also found in the FTIR analysis. In any case, the most significant difference was observed for the LD samples, with a T_g_ about one degree lower than for the remaining pieces.

### 3.5. Isothermal Crystallization Behavior

**Figure 6:**
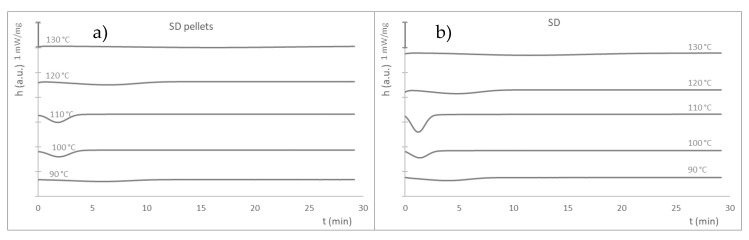
Thermograms obtained for SD materials in the isothermal crystallization test ((**a**): pellets; (**b**): injection-molded samples).

The crystallization kinetics of L105 processed under the different conditions were analyzed together with the melting behavior after such an isothermal step. All the samples showed similar behavior, with more intense peaks at 100 and 110 °C, which also took place earlier than for the other temperatures tested. Similar behavior was observed in previous studies [8]. A particularly similar behavior was found for the SD and LD materials before and after injection molding: it seems that injection molding has more influence than the drying stage in the thermal behavior of the material; that is, the injection molding step seems to result in further degradation of the polymer. In fact, the curves for the LD and SD injection-molded samples are overlapped; thereofre, only the SD is shown in this section. It appears that the optimal conditions for crystallization are in this temperature range, as otherwise recommended by the supplier (injection molding with 100 °C mold to obtain crystalline parts), who also claims a high rate of crystallization. The exothermal peaks are more intense for the injection-molded materials than for the pellets as a consequence of the further processing, which results in a higher degree of degradation, as was also observed in the dynamic tests. This document only shows the graphs for the SD materials (Figure 6), with the remaining graphs added as supplementary figures (Figures S1–S3).

**Figure 7:**
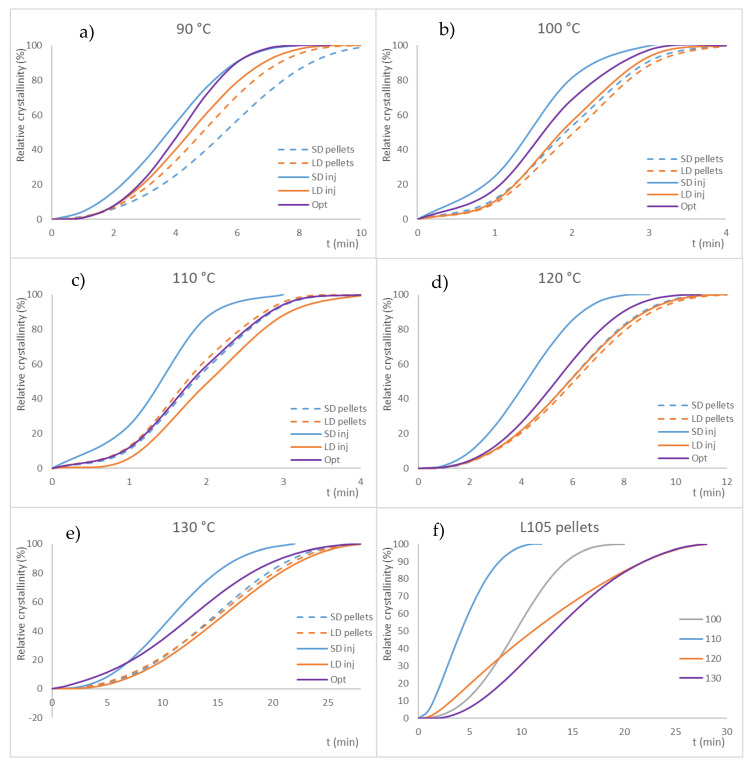
Relative crystallinity versus time obtained for the different samples in the crystallization test at the five temperatures tested (90–130 °C, (**a**–**f**)). Relative crystallinity for L105 pellets for 100 to 130 °C tests.

The materials processed under different conditions show a similar run (Figure 7), with some differences in the crystallization kinetics and extension (time and enthalpy) attributed to the various processing conditions, which result in different extents of degradation of the PLA. In this sense, it is interesting to note that the virgin pellets exhibit a much slower crystallization rate (their behavior is plotted in a separate graph for a better observation of the behavior of the remaining samples), which might confirm that the degradation occurred due to the material thermal processing.

**Figure 8:**
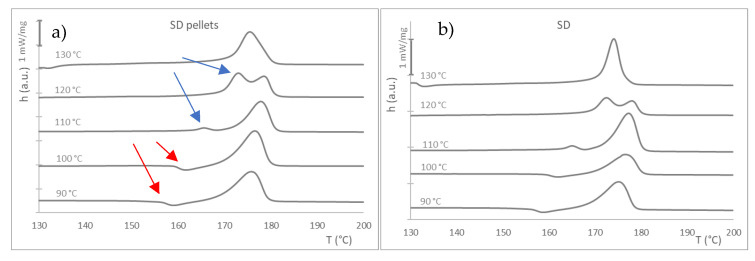
DSC curves for SD pellets (**a**) and injection-molded material (**b**) after the crystallization stage.

When considering the melting behavior after the crystallization stage, similar behavior was observed for all the materials (see Figures S4–S6). The behavior for the SD materials is shown in Figure 8 as an example. Those samples with crystallization at 90 and 100 °C show a small endothermic peak at about 160 °C (T_c_, Table 6), which is attributed, as mentioned, to the shift from α′ to α crystals (red arrows in Figure 8). Zhang and collaborators found that α′ crystals tend to form at crystallization temperatures below 100 °C and α′ and α coexist between 100 and 120 °C, while only α crystals are found over 120 °C [46]. This peak disappears and shifts into an exothermic event, which is more significant at 120 °C (blue arrows), and converges toward a single melting peak for the test at 130 °C. The curves and data obtained from experiments performed at 140 and 150 °C are not shown, as no crystallization took place for any of the samples at these temperatures.

All the samples, regardless of their processing, showed the same behavior, although with some shifts in the values of temperature and with different levels of crystallinity. This might mean that the processing (drying conditions and extrusion) results in the degradation of the polymer, although to a relatively limited extent. Apart from this, the trend of the curves for each temperature is similar. Generally, crystallinity values from melting enthalpy increase up to 100–110 °C and then are reduced again. A similar trend is obtained if calculating the crystallinity from the enthalpy of crystallization in the isothermal step of the assay. The time peak in the crystallization step follows a similar course for all the samples, except for the virgin pellets, which, again, show different behavior, with a crystallization time much higher for the lower temperature (90 °C) than the rest of samples, although with a similar tendency: a decrease of the time (faster crystallization process) up to 100–110 °C and an increase for higher temperatures.

### 3.6. Mechanical Testing

The results obtained for the mechanical testing of the injection molded samples are summarized in Table 7. As can be observed, higher processing leads to a reduction in the tensile properties, i.e., no significant differences were found in the tensile strength of L105 and Opt, while SD and LD exhibited lower values; on the other hand, the tensile modulus was significantly reduced for LD. This reduction in stiffness might be related to a certain degradation of the material during the long drying time for the LD series. Similar behavior was observed in the flexural testing, with no differences between the SD and LD series and slight changes (an increase, in this case) for the flexural strength of Opt compared to L105. Finally, no significant changes were observed in the flexural modulus and impact strength as a consequence of the processing conditions. These results also show that the mechanical performance of the PLA L105 is not affected by the conditions in which injection molding is performed (lower screw rotation speed and higher holding pressure for Opt), being that the mechanical properties are more significant than those of the pre-processed material (extrusion stage). This is important to consider when comparing the behavior of neat polymer versus composites, where a compounding step is usually performed; the results should be compared to the polymer experiencing the same processing cycles to analyze the effect of the filler.

Although not included in the previous table, the strain at ultimate strength exhibits a similar trend: close values for L105 and Opt (2.3% on average), and lower values for SD and LD (with an average of 1.7%), which are also close between them. This also reflects the increased brittleness of the materials due to the extrusion process, as was also concluded from the DMA analysis.

### 3.7. Thermomechanical Performance

**Figure 9:**
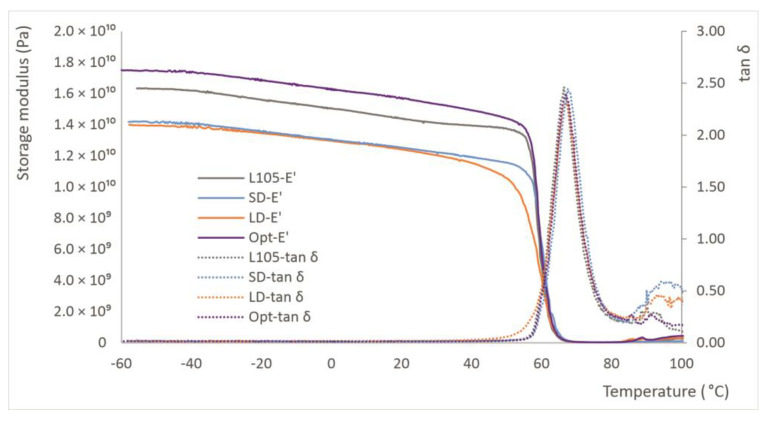
Curves for storage modulus (left axis; continuous line) and tan δ (right axis; dotted line).

Dynamic mechanical analysis (DMA) is an important test for the evaluation of the mechanical properties of materials because it is sensitive to structural changes [48]. The analysis of the storage modulus (E′), loss modulus (E″), and damping factor (tan δ) with temperature allows for an evaluation of the change in mechanical properties due to different processing of the same material along the temperature range (Figure 9). The curves follow an expected trend, with almost a plateau plotted for the storage modulus at lower temperatures and a sharp decrease after reaching a certain temperature, close to the heat deflection. The SD shows the highest E′ values, close to L105, while the Opt and LD provide lower values throughout the entire test, which were very similar to each other. However, the temperature at which the storage modulus drastically falls was different for the different series: 57 °C for L105 and Opt, 56 °C for SD, and 50 °C for LD, although this last measurement shows a higher slope from 40 °C, which represents the lower stability of the material. This region shows the change from a material that is predominantly elastic to one that is mostly viscous. This is also seen at the beginning of the tan δ peak for this material, despite the intensity of this curve being similar for the four sets of samples. This equal value for the damping factor correlates with the impact behavior, which was also found to be similar. The maximum value found in tan δ can be considered as the glass transition temperature and is found to be around 67 °C for all the samples (Table 8). The appearance of bumps after T_g_ is related to cold crystallization, as was also observed in the DSC tests. The results from the DMA correlate with static testing and confirm that the extrusion of the material is more significant than the injection-molding conditions. The longer drying time seems to significantly decrease the thermal stability of the material, as the rubbery state occurred at lower temperatures for LD than for the other sets.

Table 8 shows a summary of the storage modulus data at different temperatures, the damping factor, and the brittleness calculated at room temperature, using the equation proposed by Brostow et al. [49] (Equation (9)). According to these authors, lower values of brittleness are related to a higher dimensional stability of the material under repetitive loading:

(9) $$B=\frac{1}{\varepsilon_{b}\cdot E^{\prime }}$$

where *B* is the brittleness, *ε_b_* is the elongation at the break, and *E*′ is the storage modulus from DMA at 1.0 Hz, both at room temperature.

As previously mentioned, the storage modulus remained practically unchanged until T_g_ was reached, then it was drastically reduced. The loss modulus was also almost unchanged, with values around 100 times lower than the storage modulus, until reaching maximum values of 2.2–2.7 GPa at about 60 °C. The maximum values for the damping factor were obtained at about 67 °C. Finally, the brittleness factor (calculated at 25 °C) shows an increasing trend with the processing, especially for the LD samples, as was also observed in the static mechanical testing. This means that the reprocessing reduces the thermal stability of the injection-molded pieces. One of the main drawbacks of PLA is its low thermal resistance: the processing conditions impact the thermomechanical behavior; therefore, it is a critical issue to consider. The use of chain extenders or other additives would be needed if the PLA undergoes more than one thermal process in order to reduce the degradation of its properties.

### 3.8. Rheology

**Figure 10:**
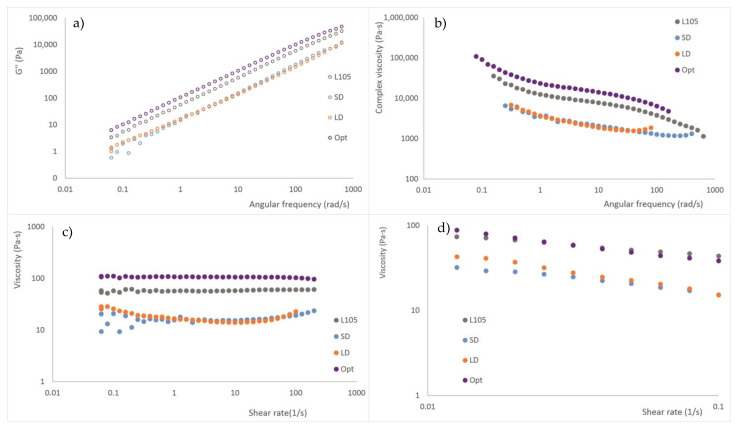
Results of frequency-sweep rheological experiments: (**a**) loss modules, (**b**) complex viscosity, (**c**) viscosity, and (**d**) viscosity at low frequencies from the flow test.

The previous analysis of the storage and loss moduli (G′ and G″) versus strain, obtained during the strain sweep assays, was performed to ensure that the analysis took place in the linear viscoelastic region (0.1–5.0% strain). We chose 0.5% strain for the frequency sweep tests. For all the ranges studied, the values of the material injected in optimal conditions are close to those of L105 (unextruded material), while SD and LD provided similar lower values. Figure 10 shows the loss modulus and viscosity obtained from the frequency sweep and flow tests in the oscillation mode. No cross-point (G′ = G″) was observed for any of the materials, and the loss modulus was higher than the storage modulus for all the studied ranges, which means that the viscous part is dominant over the elastic one. As is true for G′ and G″, the behavior of LD and SD is similar, while the course of the curves for L105 and Opt are also similar, with higher values for Opt. For these materials, the viscosity is reduced with the increase of the shear rate, reaching a plateau area from about 1 to 10 rad/s. L105 and Opt show pseudoplasticity, so the material is less viscous at increasing angular frequencies. LD and SD also show this, although, at a certain point, the viscosity tends to rise again. From the flow tests, the viscosity behavior at low shear rates provides similar observations, with even more similar behavior of the L105 and Opt materials.

L105 is a relatively low molecular weight material with high flow; that is, it has low viscosity, as was found from the zero-shear viscosity (η_0_) obtained from the Carreau–Yasuda regression in the flow test. The values for L105 and Opt were, again, quite similar (72 and 128 Pa·s), while SD and LD provided lower values (33 and 54 Pa·s, respectively). The injection pressure also varied similarly for both groups of materials, being about 510 bar for SD and LD and around 625 bar for L105 and Opt.

It can be concluded that injection-molding conditions modify the flow behavior of the material. On the other hand, the extrusion process leads to material with reduced viscosity, which could be an indication of lower molecular weight, indicating the degradation of the raw polymer, to a certain extent, due to the thermal cycle suffered by the polymer. However, the exposure to short or long processing times does not seem to influence the polymer rheology, which would reduce the restrictions for material processing. These results are in agreement with the DSC analysis (Section 3.5), where Opt and L105, and SD and LD exhibited similar behavior.

### 3.9. Color Evaluation

It is well known that the degradation of PLA is evidenced not only by changes in the mechanical, flow, or thermal properties, but also by the appearance of a yellowish color in the samples. Table 9 summarizes the average values of the color parameters, yellow index, and opacity in the injection-molded samples. As observed by the naked eye, those samples with two processing cycles show some yellowing, increasing this parameter by over 100%; this can be considered as an indicator of partial degradation of the polymer, as was also observed in the different characterization techniques employed. The color parameters are similar for all the samples, regardless of their thermal history.

### 3.10. Characterization of Biaxially Stretched Samples

A number of injection-molded plaques for each series of materials were biaxially stretched into sheets and their tensile properties were measured. A statistical analysis was used to determine the differences in the mechanical performance for a range of stretching conditions (temperature, time, and stretch ratio). The yield strength, strain at yield, strength at breakage, elastic modulus, opacity, and thickness of the stretched sheets were analyzed. In the second step, the same parameters were analyzed for the different series; that is, the influence of the material pre-processing was analyzed for the same stretching conditions.

#### 3.10.1. Processability under the Selected Conditions

In the first step, conducted at a stretch ratio of 3, the temperature, strain rate, and heating time were varied, following the Doehlert design shown in Table 10. A previous DSC analysis was used to establish the limits for the temperature and heating time. As previously mentioned, stretching should be performed between the glass transition and cold crystallization temperatures, namely, between 60 and 95 °C. Preliminary testing in this temperature range allows for the determination of the influence of the strain rate and heating time on the crystallization of the material. It was found at the higher end of the temperature range that the samples break during stretching due to excessive rigidity, with an observed loss in transparency; the crystalline parts change from transparent to white. At the lower end of the temperature range, the samples also broke due to the lower mobility of the chains (too close to T_g_). These preliminary trials allowed the limits for the first set of samples to be determined, providing the results summarized in Table 10.

Yellow indexes are very low (maximum value of 0.33 for LD series), even for samples processed by extrusion, which showed higher values for the injection-molded samples; the material stretching results in a thin sheet with almost no color for all the series. In any case, the same trend as for the injection molded samples was observed, namely, the YI is higher for LD in general terms, followed by SD. Regarding the opacity, significant variations were found for the different processing conditions. Despite the similar values for the L-105 and Opt injection-molded samples for this parameter, the stretched sheets provided lower opacity (or higher transparency) for Opt than for L105. According to the manufacturer’s datasheet, a slow injection rate is recommended (as for Opt), and this is one of the results of such conditions.

Before analyzing the stress–strain curves obtained for each set of samples, a first assessment was conducted on the basis of the process itself. As previously explained, a value of 1 was given for the samples that stretched without breaking and without whitening, 0 for those samples that broke during the process, and 0.5 for those that that showed whitening, even when stretching correctly. At first glance, it can be observed that most samples can be processed under the selected conditions. In addition, all the samples from the pellets that were not previously extruded (L105 and Opt) showed the same behavior, while those from the extruded pellets (SD and LD) showed lower processability, especially at higher temperatures. This correlates with the results from DSC analysis, which showed a faster crystallization rate and a lower cold crystallization temperature (closer to the 92.8 °C used in the experiment). The processability is correlated with the opacity for the sheets obtained: lower values of opacity (and lower deviations in the measurements) were obtained for the conditions with higher processability (marked as 1), while those with whitening provided higher values of opacity, not only on average, but also in deviation. This higher dispersion of results is due to the fading of the samples. The samples with the highest opacities were not analyzed further due to the inhomogeneities observed.

The forces recorded in both axes (x and y) during the biaxial stretching show a difference under 10% for SD and LD, and approximately 15% for L105 and Opt, as shown in Figure S7. This can be related to the lower viscosity of these materials due to their reprocessing, which results in lower resistance to the filling of the cavity. In any case, as the energy needed to stretch the injection-molded sample in both directions is close for both axes, it can be stated that the material shows a relatively isotropic behavior, and injection molding flow does not significantly influence the samples’ mechanical performance. The force needed to stretch the injection-molded preforms is similar for all the series, with only the Opt series demonstrating higher values (for the processing at a lower temperature). The maximum force recorded during the deformation is usually found at the end of the process, and its increase is attributed to the increased value of crystallinity. As explained in the introduction section, PLA is able to crystallize under strain, as is also the case with PET (strain-induced crystallization: SIC). No correlation between the different values of force required for the deformation and the properties of the samples (before or after the stretching) has been found.

#### 3.10.2. Tensile Properties of Stretched Sheets—Influence of Stretching Conditions

Table 11 shows the characterization results for L105; the same analysis was performed for the different series of materials and can be found later in this section. As can be observed, the series of tests performed at 87.5 °C with stretch ratio of 1 and a heating time of 68 s provide results with high deviations for all the properties. In addition, the opacity of these samples shows higher values, close to 5%; the crystallization of PLA results in whitening of the crystallized part, and so it can be assumed that these conditions increase the crystallinity of the samples in a non-homogeneous manner, a fact which explains the inconsistent behavior of this series. In fact, this series shows significant differences with respect to the remaining series for all the properties (except for the strain at yield strength and thickness). Finally, this series of samples exhibits the lowest properties, particularly regarding the yield and break strength, which are critical factors for the functionality of the final parts in the intended applications (packaging, for example). It is interesting to note that no significant differences were found for the samples stretched at the same temperature, meaning that the stretch ratio and heating time do not appear to have a considerable influence on the properties of the tested samples. The stretching temperature can be seen to influence the yield strength: a lower heating temperature results in higher yield strength, while higher temperatures result in lower strength. In contrast, the elastic modulus exhibits different behavior, which is dependent on the stretching conditions. Biaxial tests carried out at the lowest and the highest temperatures had similar values for the elastic modulus; the average value for the tests at 82.2 °C was 3308.5 ± 125.0 MPa, while for 92.8 °C, it was 3148.2 ± 297.1 MPa, without significant differences between them. The intermediate temperature used (87.5 °C) shows distinct behavior that varies with the heating time. The highest value for the elastic modulus was obtained after 68 s of heating, independent of the strain rate, while the lowest value was obtained after 90 s. It seems that heating times over 68 s are excessive for this temperature. It is interesting to note that the sheets broke during the stretching at 92.8 °C for times over 56 s, which confirms that an excessive heating time results in lower processability due to the development of crystallization, as was also observed in the opacity values. Despite not all the series providing significant differences in opacity, there is a clear trend toward an increase in opacity with heating, with the highest values being those of the samples heated for 90 s at 87.5 °C and for the series at 92.8 °C. Finally, no significant differences were found in the thickness or strain at yield strength, with averages values of 94 ± 14 microns and 3.1 ± 0.3% for L105.

From this analysis of the L105 samples, it can therefore be concluded that the samples processed at a lower temperature exhibit the best results, i.e., good processability, high yield and breaking strength, and high tensile modulus, at a lower energy cost. Similar results were obtained for the analysis of Opt, as shown below.

**Figure 11:**
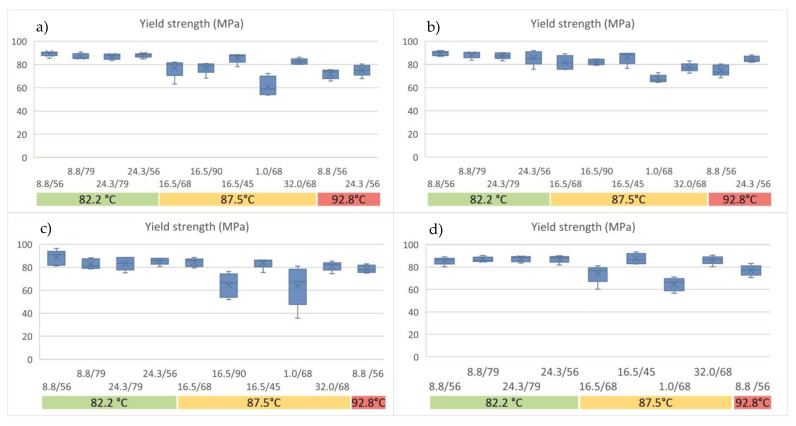
Yield strength obtained for the stretched sheets from the different injection-molded preforms: (**a**) L105, (**b**) Opt, (**c**) SD, and (**d**) LD.

For the Opt series of tests, similar conclusions arose (all the data are provided in the Supplementary Materials, Table S1); the samples obtained at a strain rate of 1 s^−1^ exhibited high standard deviations and an extremely low value for the yield strength. The low stretching speed therefore influences the performance of the material, most likely due to the partial orientation of the molecules during stretching, which results in partial crystallization of the sample, as was also observed in the higher value of opacity. This series, as well as the series heated for 90 s, resulted in higher opacities, as was also the case for L105. These conditions can then be discarded. The yield strength was only affected by the stretching temperature, but not by the heating time or strain rate (Figure 11b), with higher values demonstrated again for the lowest processing temperature: 87.8 ± 3.9 MPa for samples at 82.2 °C and 81.5 ± 5.1 MPa for the remaining two temperatures. The elastic modulus for all the series is similar in this case, without significant differences, except for the series 87.5/16.5/68, which exhibits a significantly higher value than the remaining samples (*p*-value < 0.04). The average value of the elastic modulus for Opt is 3181.6 ± 208.6 MPa (similar to the values obtained for the L105 series), whilst the maximum value is 3677.8 ± 152.9 MPa for 87.5/16.5/68.

Finally, the strength at breakage, the thickness, and the strain at yield strength do not show any significant difference between the series. For the breaking strength, it occurs the same as for the yield strength: lower temperatures provide higher values, although this difference is not statistically significant (minimum *p*-value of 0.059 for comparisons by pairs).

For the SD samples, the series 92.8/8.8/56 showed over 100% higher opacity (and deviation) than the remaining samples, so these samples were not further analyzed. LD showed low opacities for low temperatures or short times, while the higher temperatures or times over 56 s led to higher values (reduced transparency). For both SD and LD (Tables S2 and S3, respectively) and L105 and Opt, the strain at yield was consistent among all the series, around 3%. It was also seen that the strain at yield was unaffected by the thermal history of the material (extruded vs. non-extruded) or by the stretching conditions.

The thickness only differs significantly (*p* < 0.035) for the series 87.5/16.5/90 for SD, which had an average value of 121 μm, while the remaining ones exhibited an average thickness of 84 μm. This series of samples also had relatively high opacity, which results in lower mechanical performance. As observed for Opt and L105, the heating time of 90 s was too long, and the cold crystallization of the material occurred, to a greater extent, due to the faster kinetics of the samples extruded. Furthermore, the SD injection-molded samples did not result in the obtaining of sheets under these conditions (breakages during the stretching). Despite the higher temperatures leading to higher opacity, the remaining series were analyzed in order to assess the influence of such variables in the final behavior of the stretched sheets.

For SD, the yield strength was not affected by the temperature, contrary to what was observed for the unextruded materials (Figure 11c); it seems that reprocessing the materials (as may be derived from recycled material) delivers more consistent behavior, regardless of the stretching conditions. Only the samples stretched at a low strain rate (1 s^−1^) provided a significantly lower yield strength. The results for the yield strength follow a similar trend to those for the opacity, as well as for LD. Lower temperatures or shorter heating times lead to higher yield strength (Figure 11d). Many of the series showed significantly different behavior in the elastic modulus either for SD or LD. The breaking strength does not show any clear trend with the stretching conditions, although there was a trend for obtaining lower values with a low strain rate; the behavior of the samples at the same temperature provided comparable values.

From this analysis, it can be inferred that, once again, the behavior of the stretched series of the L105 and Opt series are similar between, the same of which is also true for SD and LD. The strain at yield is not affected by the stretching conditions for any of the series. The yield strength shows a negative trend with temperature, particularly for the unextruded samples: higher temperatures lead to lower resistance. The yield strength shows the same trend as the opacity values: higher transparency (lower opacity) usually leads to higher yield strength. The tensile modulus and strength at break seem to provide an unclear trend with the processing condition, although it seems that higher stretching temperatures results in lowered values.

#### 3.10.3. Stretching Parameters Optimization: Surface Response Curves

From this first set of experiments, it can be seen that the highest temperature does not provide satisfactory results for all the samples due to the fast crystallization of the material. A reduced processing temperature without an increase in the processing time would be beneficial, as this leads to a reduction in energy costs. Separate regressions were performed in order to obtain a relationship between the different parameters in the process (temperature, time, stretch ratio, and processing conditions) and the response of the material (yield or breaking strength, modulus, opacity, and force). The analysis was performed for each material, and the results were combined with those of the extruded (SD and LD) and non-extruded (L105 and Opt) materials; the results obtained from all the tests were close for these sets (results not shown). Table 12 summarizes the value for the adjustment coefficient (R^2^) and optimal conditions for each comparison performed. As determined from the statistical analysis, the processing temperature is the parameter with the most decisive influence. Only for the extruded materials was a relationship between the force recorded during the stretching and the temperature and strain rate observed. In general, the materials provided similar values for optimal processing: a higher temperature (around 90 °C) in order to minimize the force during stretching, and 82 °C to maximize the yield strength. The force needed during stretching is considered less important than the material performance; therefore, the conditions leading to a higher yield strength were used for increasing the stretch ratio.

**Figure 12:**
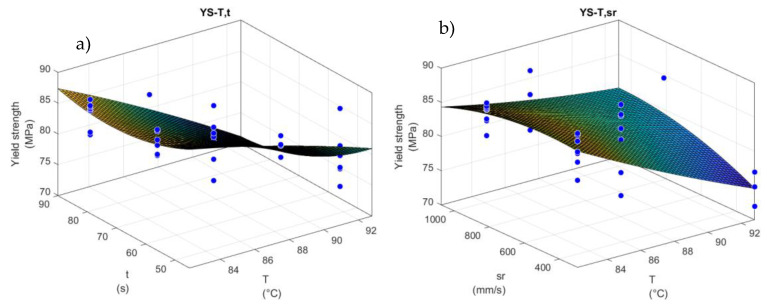
Response (yield strength) surface obtained for all series for (**a**) time and temperature, and (**b**) strain ratio and temperature.

The parameters for the equation of the surface obtained showed, in all cases, the higher relevance of the temperature, as can also be observed from the graphs below (Figure 12). Therefore, temperature has a greater influence than time, which has a more significant influence than the strain rate.

In light of this, the tests to increase the stretch ratio of the samples were performed under the optimal conditions, namely, 82 °C. A heating time of 56 s was used, as the heating time dramatically influences the energy consumption, productivity, and economy of the process.

#### 3.10.4. Tensile Properties of Stretched Sheets—Influence of Processing Conditions

As shown in the previous section, the best mechanical properties (higher and more consistent) were obtained for the materials processed at a lower temperature. It was also shown that the stretch ratio and time have very limited or no influence on the final properties of the stretched sheet, with temperature being the most critical parameter. This section, therefore, shows a comparison between the different series of materials and the properties obtained from samples stretched at 82 °C (average values and standard deviation summarized in Table 13; summary of statistics values provided in Table S4).

The statistical analysis shows no significant difference in the yield strength, strain at yield strength, or elastic modulus (Table S4). The strength at break is only different for the series with a higher value (Opt) versus the SD and LD. This analysis shows that, despite the different mechanical behavior observed for the injection-molded samples, the stretched sheets exhibit similar properties; therefore, the long drying time or the extrusion does not affect the mechanical properties of the stretched sheets to a great extent. The partial degradation of SD and LD during extrusion is reflected in the lowered strength at break, reduced by about 10% with respect to the virgin material. This is not unremarkable, mainly because of the need to make advances in the recycling of PLA post-consumer fractions. The changes in regulations banning the use of certain polymers in single-use products, particularly within the packaging industry, have increased the use of PLA in such applications due to its biodegradable character. However, it should be noted that recycling is a more favorable option than biodegradation, and the reprocessability of PLA should be further studied. Some studies have already been performed to determine the changes in properties due to recycling [9,50,51], although thermoforming or blow-molding of such materials has not yet been investigated in detail (simulated in this work with biaxial stretching tests).

#### 3.10.5. Tensile Properties of Stretched Sheets—Influence of the Stretch Ratio

This section summarizes the properties of the samples stretched under the defined optimal conditions (82 °C, 56 s) with an increased stretch ratio of 4, that is, with a deformation of 100.5 mm. The same analysis was performed, firstly assessing the processability and opacity, followed by the mechanical characterization of the obtained sheets (which can be considered as films in this case, as they are under 100 μm thick). The four series of materials were processable under these conditions and at a strain rate of 8.8 s^−1^; injection-molded PLA is stretchable, at least until a ratio of 4, regardless of its prior processing. For this considerable stretch, higher rates do not provide good results, and the samples were broken during the process. A stretch rate of 8.8 s^−1^ (294.8 mm/s) can be selected as optimal, as it allowed the stretched sheets to be obtained in a consistent way, with no failures or problems during the process. The strain-induced crystallization is evident when comparing the stretching at ratios 3 and 4. The opacity increased for all the samples (Table S5), particularly for L105, for which a completely white film was obtained. Despite this increase, the deviations obtained from these measurements are still low, which is related to the good homogeneity of the obtained samples. Regarding the force needed in the stretching process, as observed in the tests at a stretch ratio of 3, the forces in both axes are similar. The maximum values were recorded for the unextruded samples and, in particular, for L105. The lower strength required to stretch SD and LD is correlated with the reduced storage modulus found for the injection-molded samples of these materials. The increase of force for all the materials is related to the higher crystallinity of the materials obtained with the higher stretching ratio.

The samples obtained were approximately 50 microns thick, with no difference due to the processing of the samples, as was also observed for SR = 3. The thickness for SR = 4 was nearly half, and the stretching was also almost twice (SR = 3 means a deformation of 67 mm, while SR = 4 implies a deformation of 100.5 mm). The samples were homogeneous in thickness and led to consistent mechanical results with low deviations (Table 14). As observed, the properties assessed remained at similar levels, regardless of the stretch ratio, except for the elastic modulus, which increased by about 10% for all the series except L105. It is also interesting to note that, for this stretch ratio, the yield strength and strength at break took place at similar levels, with reduced (or no) plastic deformation, possibly due to the reduced thickness of the films and their higher tenacity.

The statistical analysis (Table S6) allows us to conclude that, for L105, the yield strength was reduced as a consequence of the increase in the stretch ratio; this is likely to be due to an increased crystallinity of the final film, as inferred from the white color of the film. Nevertheless, the remaining series, which did not lead to such high values of opacity, showed no significant differences in any of the assessed properties, except for the tensile modulus, which was also increased. It can therefore be concluded that higher deformation results in stiffer material, possibly due to the partial orientation of the PLA chains in the two directions of the stretching.

When comparing the different series of materials obtained for a stretch ratio of 4 (Table 15), similar conclusions arise. Only the tensile modulus of the L105 series was significantly lower compared to the remaining samples, and the opacity was higher for this series. The remaining parameters, including the stretching force, showed no statistical difference among the series, which means that the pre-processing of the material does not affect these properties in the stretched films.

#### 3.10.6. Thermomechanical Properties

**Figure 13:**
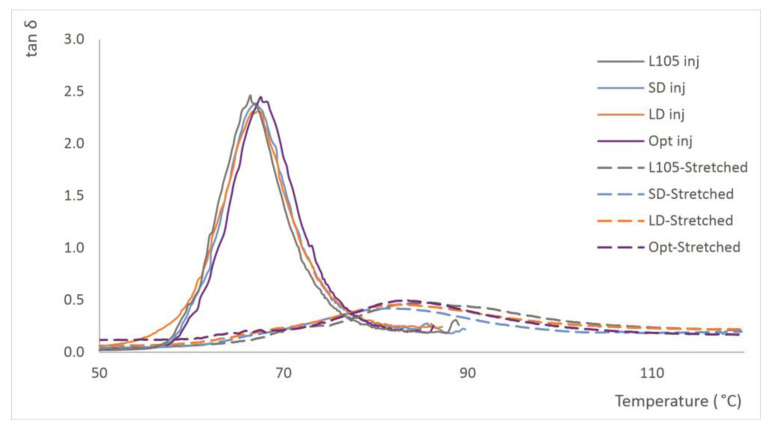
Curves for tan δ of injection-molded samples (continuous line) and samples stretched under optimal conditions (82 °C and 56 s; dashed line).

The DMA analysis shows an increase in glass the transition temperature as a consequence of stress-induced crystallization, as was also suggested by Tsai et al. [8]. For all the samples, the value increased from about 67 °C for the injection-molded samples to around 83 °C (Figure 13). This increase is also in line with the findings of Yu et al. [26], which, apart from higher crystallinity and higher T_g_, demonstrated increases in the elastic modulus of stretched samples, as was also found in this research. The increased rigidity of the stretched samples explains the lower values for tan δ found for the stretched samples.

The analysis performed for the samples stretched under different conditions (temperature, time or stretch ratio) does not provide significant differences in the glass transition temperature, since this parameter is more greatly impacted by the stretching process than by the conditions in which this is performed. As is recommended in other studies, the use of DMA is more convenient than the determination of the heat deflection temperature (HDT), as this test provides a clearer picture on the behavior of material within a wide range of temperatures, while HDT only reveals the behavior at a specific point [52]. Table 16 summarizes some of the values for the storage and loss modulus, and the temperatures for the maximum values. It was observed that higher values were obtained for the injection-molded series at a low injection speed and without processing, showing once again the impact of processing in the thermomechanical behavior of PLA.

## 4. Conclusions

The characterization of a PLA-grade (Luminy L105) sample processed in different conditions was performed to determine the crystallization kinetics, flow behavior, and mechanical properties, and it was found that, as expected, this material is susceptible to thermal processing. Some properties, such as the impact resistance or moisture uptake, were not significantly modified, while some others, such as the elastic modulus or thermomechanical behavior, showed dependency on the processing history of the material. The extrusion of the material has been demonstrated to have more of an impact than the drying conditions: the shear stress suffered during extrusion seems to produce more damage to the polymer than subjecting it to relatively high temperatures for a long time. Of particular relevance is the difference in the crystallization kinetics due to reprocessing, which correlated with a very significant increase in the MFI due to the extrusion of the materials.

It has been observed that the extrusion of the polymer (its recycling in optimal conditions) leads to degradation of the material. Although this is not visible on FTIR, the higher MFI of the extruded materials compared to the virgin ones, and the increased crystallinity might be an indication of the beginning of the degradation. The increase in the yellowness index correlates well with these observations. Finally, the mechanical behavior and rheology of the materials are also affected by reprocessing; that is, the extrusion step results in lower viscosity (related to lower molecular weight of the polymer, due to the chain scissions), and also in lower mechanical properties, and is particularly relevant to the flexural behavior. On the other hand, the modification of injection molding parameters does not modify, to a great extent, the mechanical properties of the samples, while those parts produced under optimal conditions (lower feed rates and higher holding pressure) exhibit a higher value of the storage modulus, and also provide more stable behavior within the temperature range studied. This is particularly relevant for this material, as one of its main drawbacks is the low heat deflection temperature, which limits its application window. In this sense, the stretching of the PLA injection-molded samples appears as an interesting option, not only because the mechanical properties of the films are good for packaging applications (and are consistent, with low deviations), but also because their thermal stability is increased due to the orientation of the polymer chains during the process. The maximum values for tan δ can be achieved at roughly 67 °C for injection-molded samples, improving upon the 80 °C requirement for the stretched samples.

The operation window to perform the cold-stretching process was determined with the crystallization tests and placed between 80 and 95 °C, that is, about 20 °C over the glass transition temperature, and 10 °C below cold crystallization.

The injection-molded test plaques were then subjected to biaxial stretching at a stretch ratio of 3, and it was determined that the temperature had a more substantial influence than time or the strain rate. A temperature of 82 °C was found to provide the optimal conditions: good processability of all the samples, with no failures, and good mechanical properties of the stretched films, particularly in terms of the yield and breaking strength.

Despite the different properties obtained for the injection-molded biaxial test plaques, the behavior of the stretched films was more uniform among the series. The stretch ratio only affected the elastic modulus of the films, bringing higher values for SR = 4 than for SR = 3. The average values for the yield and breaking strength for these films were about 90 MPa, with an elastic modulus around 4000 MPa, which shows the high potential of this PLA grade for use in the manufacture of sheets and films through blow-molding or thermoforming. Some additional testing would be needed to ensure its appropriateness for food-packaging applications, such as barrier properties or properties at different temperatures (e.g., for frozen food).

## Figures and Tables

**Table 1:** Processing conditions for PLA Luminy L105 from Corbion Polymers.

Short Name	Drying	Processing
L105 pellets	None	None
SD pellets	75 °C 8 h + 100 °C 4 h	Extrusion
LD pellets	75 °C 8 h + 100 °C 4 h 75 °C 48 h + 100 °C 12 h	Extrusion
L105 inj	75 °C 8 h + 100 °C 4 h	Injection molding
SD inj	75 °C 8 h + 100 °C 4 h	Extrusion + injection molding
LD inj	75 °C 8 h + 100 °C 4 h 75 °C 48 h + 100 °C 12 h	Extrusion + injection molding
Opt	75 °C 8 h + 100 °C 4 h	Injection molding (same material as L105; injection-molded at lower flow rate and higher holding pressure)

**Table 2:** Experiments performed on PLA-injection molded samples, following a Doelhert design of experiments.

Short Name	Temperature (°C)	Stretch Ratio	Strain Rate (s^−1^)	Strain Rate (mm/s)	Time (s)
82.2/8.8/56	82.2	3	8.8	294.8	56
82.2/8.8/79	82.2	3	8.8	294.8	79
82.2/24.3/79	82.2	3	24.3	814.1	79
82.2/24.3/56	82.2	3	24.3	814.1	56
87.5/16.5/68	87.5	3	16.5	552.8	68
87.5/16.5/90	87.5	3	16.5	552.8	90
87.5/16.5/45	87.5	3	16.5	552.8	45
87.5/1.0/68	87.5	3	1.0	33.5	68
87.5/32.0/68	87.5	3	32.0	1072.0	68
92.8/8.8/56	92.8	3	8.8	294.8	56
92.8/8.8/79	92.8	3	8.8	294.8	79
92.8/24.3/79	92.8	3	24.3	814.1	79
92.8/24.3/56	92.8	3	24.3	814.1	56
82.2/8.8/56/4	82.2	4	8.8	294.8	56

**Table 3:** Absorbance ratios for characteristic bands in PLA degradation.

Sample	Absorbance Ratios for Different Wavelengths (in cm^−1^)					
	1747/1465	1207/1465	1183/1465	1085/1465	920/1465	756/866
L105 pellets	0.876 ± 0.049	0.952 ± 0.021	0.885 ± 0.043	0.861 ± 0.056	1.042 ± 0.019	0.989 ± 0.005
SD pellets	0.715 ± 0.124	0.883 ± 0.051	0.740 ± 0.107	0.664 ± 0.138	1.101 ± 0.044	0.993 ± 0.003
LD pellets	0.679 ± 0.098	0.872 ± 0.048	0.709 ± 0.092	0.632 ± 0.122	1.123 ± 0.030	0.988 ± 0.009
L105 inj	0.429 ± 0.135	0.758 ± 0.050	0.419 ± 0.107	0.267 ± 0.124	1.206 ± 0.059	0.985 ± 0.001
SD inj	0.423 ± 0.237	0.767 ± 0.091	0.454 ± 0.217	0.323 ± 0.261	1.209 ± 0.086	0.980 ± 0.005
LD inj	0.542 ± 0.226	0.798 ± 0.090	0.523 ± 0.208	0.387 ± 0.254	1.167 ± 0.087	0.984 ± 0.004
Opt	0.456 ± 0.028	0.763 ± 0.013	0.441 ± 0.024	0.288 ± 0.028	1.202 ± 0.014	0.984 ± 0.003

**Table 4:** Water uptake parameters.

Material	D (m^2^/s)	k (%/s^0.5^)	n	R^2^	Swelling (%)	Sorption (g/g)	Permeability (m^2^/s)
L105	7.46·10^−14^	5.34·10^−2^	0.23	0.923	0.07	0.006	4.80·10^−16^
SD	1.01·10^−13^	2.51·10^−2^	0.27	0.919	0.16	0.007	6.94·10^−16^
LD	9.35·10^−14^	2.48·10^−2^	0.27	0.925	0.11	0.007	6.68·10^−16^
Opt	1.23·10^−13^	1.78·10^−2^	0.29	0.900	0.07	0.007	8.17·10^−16^

**Table 5:** Thermal parameters for PLA samples from DSC tests for the second heating cycle (T_m_: melting temperature, T_cc_: cold crystallization temperature, T_s_: solidification temperature, T_g_: glass transition temperature, χ: crystallinity degree).

Sample	T_m2_ (°C)	T_cc2-1_ (°C)	T_cc2-2_ (°C)	T_s_ (°C)	χ_2_ (%)	T_g2_ (°C)
L105 pellets	172.94	--	--	102.41	14.4	59.65
Pellets SD	175.87	99.45	159.31	94.99	7.6	59.47
Pellets LD	174.91	99.89	158.65	95.91	7.6	59.18
L105 inj	174.37	100.67	157.42	95.73	6.0	60.42
SD inj	175.18	96.55	158.82	96.54	13.3	60.03
LD inj	173.90	94.54	158.54	97.83	11.1	58.67
Opt	173.88	100.22	156.69	94.73	8.3	59.86

**Table 6:** Thermal parameters obtained for the isothermal crystallization tests (T_cryst_: temperature of the crystallization stage; T_c_: exothermal peak related to α′ to α transition; T_m_: melting temperature; T_m2_: melting temperature of the secondary melting peak; χ_m_: crystallinity calculated from the melting enthalpy in the heating curve; t_peak_: time of the minimum found in the crystallization stage). Blank spaces indicate peaks not appearing in the corresponding thermogram.

Sample	T_cryst_ (°C)	T_c_ (°C)	T_m_ (°C)	T_m2_ (°C)	χ_m_ (%)	t_peak_ (min)
L105 pellets	90	155.8	174.3		16.5	
	100	158.9	174.7		13.1	22.1
	110		170.3	164.1	18.1	2.8
	120		177.4	171.4	13.4	5.2
	130		170.0		15.7	12.9
SD pellets	90	157.7	174.4		15.3	5.9
	100	160.2	175.0		16.1	1.9
	110		176.3	164.0	13.7	1.9
	120		177.2	171.5	17.0	6.4
	130		174.0		17.2	16.2
LD pellets	90	158.0	175.2		15.2	5.9
	100	160.2	174.8		16.9	2.0
	110		176.9	166.3	12.5	1.8
	120		177.2	171.4	12.0	6.7
	130		173.7		15.0	15.8
L105 inj	90	156.0	174.2		15.8	5.0
	100	159.0	174.2		13.6	1.9
	110		175.6	163.8	11.2	2.0
	120		176.4	170.4	13.0	6.8
	130		172.7		10.8	15.3
SD inj	90	157.4	173.6		18.2	3.9
	100	160.5	175.1		13.2	1.4
	110		175.6	166.3	22.1	1.2
	120		176.8	171.1	15.7	4.8
	130		172.6		21.5	11.3
Opt	90	155.4	173.4		13.8	4.6
	100	158.4	173.4		14.3	−0.8
	110		174.6	163.3	13.0	1.8
	120		176.4	170.0	15.0	5.9
	130		172.4		13.6	12.2

**Table 7:** Average values (±standard deviations) for mechanical properties of the materials.

Material	Tensile Properties		Flexural Properties		Impact
	Strength (MPa)	Modulus (MPa)	Strength (MPa)	Modulus (MPa)	Strength (kJ/m^2^)
L105	48.65 ± 5.35 ^a^	2378.47 ± 72.61 ^b^	93.40 ± 0.98	2757.56 ± 100.54 ^d^	7.37 ± 0.38 ^e^
SD	37.34 ± 2.59	2207.87 ± 12.90 ^b^	67.43 ± 1.20 ^c^	2753.41 ± 65.29 ^d^	7.01 ± 0.22 ^e^
LD	33.44 ± 1.78	2078.41 ± 161.89	65.56 ± 1.85 ^c^	2750.47 ± 237.41 ^d^	6.96 ± 0.43 ^e^
Opt	49.55 ± 3.26 ^a^	2158.78 ± 78.29 ^b^	98.20 ± 0.56	2636.89 ± 125.08 ^d^	6.98 ± 0.13 ^e^

Tukey tests for the comparison of properties of the different series of materials have been used at a 95% confidence level. Those materials with the same superscript letter show no statistical difference for the property.

**Table 8:** Average values (± standard deviations) for mechanical properties of the materials at room temperature.

Material	E′ (10 ^9^ Pa)				E″ (10^9^ Pa)			tan δ		B (10^12^ Pa^−1^%^−1^)
	−20 °C	0 °C	25 °C	65 °C	25 °C	65 °C	Maximum (T)	25 °C	Maximum (T)	
L105	15.60	15.04	14.23	0.28	0.25	0.62	2.51 (59.4 °C)	0.0174	2.46 (66.4 °C)	30.55
SD	13.53	13.02	12.38	0.63	0.19	1.07	2.46 (61.1 °C)	0.0152	2.45 (67.5 °C)	35.79
LD	13.45	12.97	12.25	0.37	0.24	0.71	2.20 (60.0 °C)	0.0192	2.31 (67.3 °C)	48.03
Opt	16.89	16.28	15.52	0.40	0.23	0.79	2.69 (59.9 °C)	0.0148	2.38 (67.0 °C)	33.66

**Table 9:** Parameters for color and gloss evaluation.

Material	Color Parameters				Yellow Index	Opacity (%)
	L*	a*	b*	ΔE		
L105	88.58 ± 0.03	−1.03 ± 0.02	1.06 ± 0.02	88.60 ± 0.03	1.71 ± 0.04	2.41 ± 0.16
SD	88.39 ± 0.02	−1.20 ± 0.00	2.08 ± 0.03	88.42 ± 0.02	3.36 ± 0.04	1.87 ± 0.15
LD	88.12 ± 0.05	−1.22 ± 0.02	2.41 ± 0.07	88.16 ± 0.05	3.91 ± 0.11	1.96 ± 0.19
Opt	88.44 ± 0.13	−1.06 ± 0.02	1.13 ± 0.08	88.46 ± 0.12	1.82 ± 0.13	2.24 ± 0.33

**Table 10:** Results of the first trials on PLA-injected samples for a stretch ratio of 3.

Short Name	Processability				Opacity (%)				Maximum Force (N)			
	L105	SD	LD	Opt	L105	SD	LD	Opt	L105	SD	LD	Opt
82.2/8.8/56	1	1	1	1	2.45 ± 0.33	0.43 ± 0.15	0.36 ± 0.14	0.93 ± 0.24	45.443	20.269	22.328	79.871
82.2/8.8/79	1	1	1	1	3.12 ± 0.10	0.96 ± 0.17	1.15 ± 0.17	1.82 ± 0.07	35.854	28.659	27.356	40.561
82.2/24.3/79	1	1	1	1	2.74 ± 0.12	1.25 ± 0.26	0.79 ± 0.09	2.68 ± 0.94	49.723	36.723	38.220	90.306
82.2/24.3/56	1	1	1	1	2.72 ± 0.66	0.78 ± 0.05	0.65 ± 0.06	2.25 ± 0.77	61.890	39.410	42.400	104.207
87.5/16.5/68	1	1	1	1	2.20 ± 0.31	3.91 ± 0.49	6.74 ± 1.27	1.65 ± 0.21	12.989	26.145	25.304	42.000
87.5/16.5/90	1	1	0	1	4.43 ± 1.73	8.04 ± 1.74	--	3.53 ± 0.40	28.477	28.385	--	29.682
87.5/16.5/45	1	1	1	1	1.75 ± 0.13	0.64 ± 0.15	0.24 ± 0.07	0.74 ± 0.10	26.974	30.059	29.359	45.026
87.5/1.0/68	1	1	1	1	4.83 ± 0.27	5.99 ± 0.40	7.51 ± 0.14	3.91 ± 0.75	36.127	14.607	11.341	17.083
87.5/32.0/68	1	1	1	1	1.87 ± 0.22	6.05 ± 0.97	2.06 ± 0.49	2.48 ± 0.14	33.238	31.814	29.994	31.532
92.8/8.8/56	1	0.5	1	1	3.83 ± 0.59	14.76 ± 8.86	5.86 ± 0.99	2.83 ± 0.68	20.563	16.264	20.038	21.783
92.8/8.8/79	0.5	0	0	0.5	15.09 ± 15.66	--	--	12.16 ± 10.98	22.187	--	--	20.949
92.8/24.3/79	0.5	0	0	0.5	13.26 ± 15.17	--	--	10.69 ± 9.27	29.298	--	--	28.299
92.8/24.3/56	1	1	0	1	3.22 ± 0.77	6.93 ± 1.63	--	2.87 ± 0.23	25.259	24.378	--	26.323

**Table 11:** Summary of results for the characterization of L105 samples after biaxial stretching, for SR = 3 (first column: average value; second column: standard deviation).

Short Name	Yield Strength (MPa)		Tensile Modulus (MPa)		Strength at Break (MPa)		Thickness (mm)		Strain at Yield Strength (mm/mm)	
82.2/8.8/56	89.41	2.28	3352.64	118.05	76.05	1.70	0.093	0.011	0.033	0.001
82.2/8.8/79	86.93	2.42	3230.39	120.90	107.61	5.76	0.100	0.011	0.034	0.002
82.2/24.3/79	86.97	2.46	3301.85	148.63	102.06	6.34	0.101	0.011	0.032	0.002
82.2/24.3/56	87.97	1.88	3349.21	106.88	103.15	5.67	0.096	0.013	0.031	0.001
87.5/16.5/68	76.87	7.81	3678.48	63.85	83.38	13.85	0.092	0.009	0.029	0.005
87.5/16.5/90	77.34	5.18	3066.71	155.29	87.80	6.58	0.105	0.008	0.031	0.002
87.5/16.5/45	85.67	4.32	3414.98	152.26	105.58	5.87	0.095	0.017	0.031	0.002
87.5/1.0/68	61.04	8.61	2981.71	632.08	31.74	44.89	0.100	0.022	0.027	0.003
87.5/32.0/68	82.64	2.46	3730.37	162.73	103.98	7.29	0.094	0.015	0.031	0.003
92.8/8.8/56	71.92	4.08	3213.30	144.05	84.37	10.59	0.085	0.015	0.031	0.003
92.8/24.3/56	75.02	4.77	3330.12	176.41	83.99	15.94	0.086	0.009	0.028	0.003

**Table 12:** Summary of adjustment coefficient and optimum values for different response–surface curves (response vs. parameter 1, parameter 2).

	Non-extruded		Extruded		All Series	
	R^2^	Optimum	R^2^	Optimum	R^2^	Optimum
F vs. T, sr	0.6622	90 °C	0.8809	93 °C	0.4092	90 °C
F vs. T, t	0.6062	90 °C	0.4092	--	0.3661	--
F vs. sr, t	0.0633	--	0.6460	250 mm/s, 52 s	0.0561	--
YS vs. T, sr	0.7717	82 °C	0.7669	82 °C	0.6119	82 °C
YS vs. T, t	0.7599	82 °C	0.7721	85 °C	0.6723	82 °C
YS vs. sr, t	0.0243	--	0.2304	--	0.0342	--
YS vs. T, Opacity	0.8033	--	0.8509	--	0.6369	--
YS vs. t, Opacity	0.4824	--	0.8015	--	0.3932	--

**Table 13:** Summary of mechanical properties for PLA stretched samples at 82 °C (first column: average value; second column: standard deviation).

Short Name	Yield Strength (MPa)		Tensile Modulus (MPa)		Strength at Break (MPa)		Opacity (%)		Strain at Yield Strength (mm/mm)	
L105	87.819	2.329	3342.582	101.150	98.328	98.328	2.750	0.412	0.032	0.002
Opt	87.810	3.824	3331.330	67.984	103.423	103.423	1.920	0.857	0.032	0.002
SD	84.945	5.179	3335.722	297.720	89.777	89.777	0.856	0.343	0.032	0.003
LD	86.622	2.717	3505.580	131.599	90.605	90.605	0.739	0.316	0.032	0.002

**Table 14:** Summary of results for the characterization of stretched samples for SR = 4 (first column: average value; second column: standard deviation).

Material	Stretch Ratio	Yield Strength (MPa)		Tensile Modulus (MPa)		Strength at Break (MPa)		Thickness (mm)		Strain at Yield Strength (mm/mm)	
L105	3	89.409	2.284	3352.640	118.045	76.045	1.696	0.093	0.011	0.033	0.001
	4	72.698	11.479	3444.481	254.404	71.370	3.502	0.056	0.007	0.027	0.008
SD	3	88.465	6.488	3673.083	230.223	96.269	6.428	0.081	0.014	0.032	0.003
	4	79.814	11.165	4087.793	212.442	85.651	14.812	0.046	0.007	0.025	0.004
LD	3	85.426	3.293	3494.752	148.087	102.007	12.599	0.083	0.015	0.031	0.002
	4	88.553	8.591	4250.836	195.593	84.338	7.268	0.047	0.010	0.029	0.009
Opt	3	89.631	1.984	3254.995	50.954	103.659	7.831	0.124	0.016	0.033	0.002
	4	93.638	3.380	4040.900	170.216	89.046	11.611	0.057	0.004	0.033	0.004

**Table 15:** Summary of statistics (*p*-value and mean difference) for comparison of the PLA processed under optimal conditions and SR = 4.

Comparison		Tensile Modulus		Strength at Break		Opacity		Thickness		Force	
		*p*	Difference	*p*	Difference	*p*	Difference	*p*	Difference	*p*	Difference
L105-	Opt	0.002	−596.419	0.374	−17.676	<0.001	39.453	0.994	−0.001	0.602	16.221
	SD	0.002	−643.312	0.400	−14.282	<0.001	40.000	0.192	0.010	0.118	37.837
	LD	<0.001	−806.354	0.605	−12.968	<0.001	39.977	0.399	0.008	0.074	44.397
Opt	SD	0.987	−46.893	0.984	3.395	0.609	0.547	0.126	0.011	0.409	21.617
	LD	0.419	−209.936	0.973	4.708	0.639	0.523	0.283	0.009	0.246	28.176
SD	LD	0.662	−163.042	0.999	1.314	1.000	−0.023	0.959	−0.002	0.947	6.560

**Table 16:** Average values (± standard deviations) for tensile properties of the stretched samples at different temperatures.

Material	E′ (10^9^ Pa)				E″ (10^9^ Pa)			tan δ
	−20 °C	0 °C	25 °C	65 °C	25 °C	65 °C	Maximum (T)	Maximum (T)
L105	1.73	1.80	1.78	1.37	0.05	0.14	0.26 (78.4 °C)	0.47 (85.7 °C)
SD	1.16	1.18	1.30	0.68	0.04	0.09	0.09 (64.7 °C)	0.42 (81.9 °C)
LD	1.42	1.38	0.39	0.19	0.02	0.03	0.15 (78.3 °C)	0.46 (83.0 °C)
Opt	3.63	3.69	3.69	0.91	0.14	0.16	0.38 (80.0 °C)	0.50 (83.1 °C)

## Data Availability

The data presented in this study are available from the corresponding author upon reasonable request.

## Supplementary Materials

The following supporting information can be downloaded at: https://www.mdpi.com/article/10.3390/ma16145114/s1, Figure S1: Thermograms obtained for L105 materials in the isothermal crystallization assay (left: pellets; right: injection-molded samples); Figure S2: Thermograms obtained for LD materials in the isothermal crystallization assay (left: pellets; right: injection-molded samples); Figure S3: Thermograms obtained for injection-molded Opt series in the isothermal crystallization assay; Figure S4: DSC thermograms for L105 materials after the crystallization stage (left: pellets; right: injection-molded samples); Figure S5: DSC thermograms for LD materials after the crystallization stage (left: pellets; right: injection-molded samples); Figure S6: DSC thermograms for Opt materials after the crystallization stage (left: pellets; right: injection-molded samples); Figure S7: Relationship between force recorded in the x and y axes during stretching; Table S1: Summary of results for the characterization of Opt samples after biaxial stretching (first column: average value; second column: standard deviation); Table S2: Summary of results for the characterization of SD samples after biaxial stretching (first column: average value; second column: standard deviation); Table S3: Summary of results for the characterization of LD samples after biaxial stretching (first column: average value; second column: standard deviation); Table S4: Summary of statistics (*p*-value and mean difference) for the comparisons of the PLA processed in different conditions and stretched at 82 °C; Table S5: Opacity and maximum forces recorded for PLA injection-molded samples at stretch ratios of 3 and 4; Table S6: Summary of statistics (*p*-value and mean difference) for the comparisons of the PLA processed under optimal conditions at stretch ratios (SR) of 3 and 4.
