# Supplementary material for: Characterization of PLA Sheets Prepared by Stretching under Different Conditions: Influence of Reprocessing and Establishing Optimal Conditions
Source: Materials (Basel). 2023 Jul 20;16(14):5114. doi: 10.3390/ma16145114 (PMC10386445; doi:10.3390/ma16145114)
Supplement: Supplementary file 1 [file materials-16-05114-s001.zip › materials-2516078-supplementary.pdf]

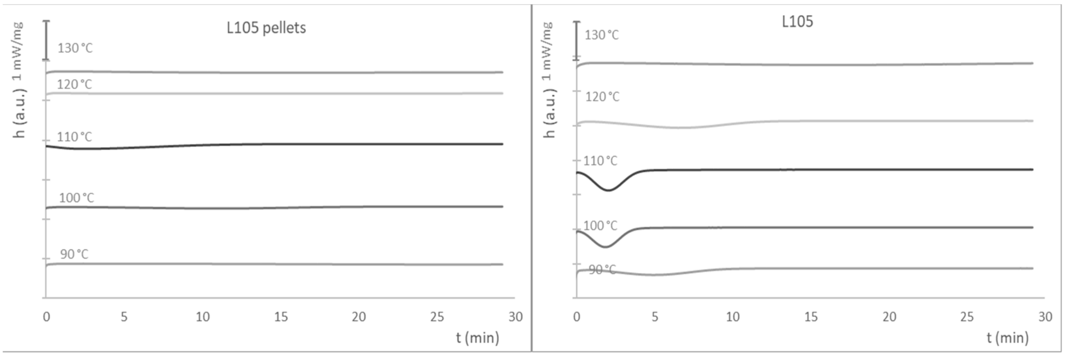

Figure S1. Thermograms obtained for L105 materials in the isothermal crystallization assay (left: pellets; right: injection-molded samples)

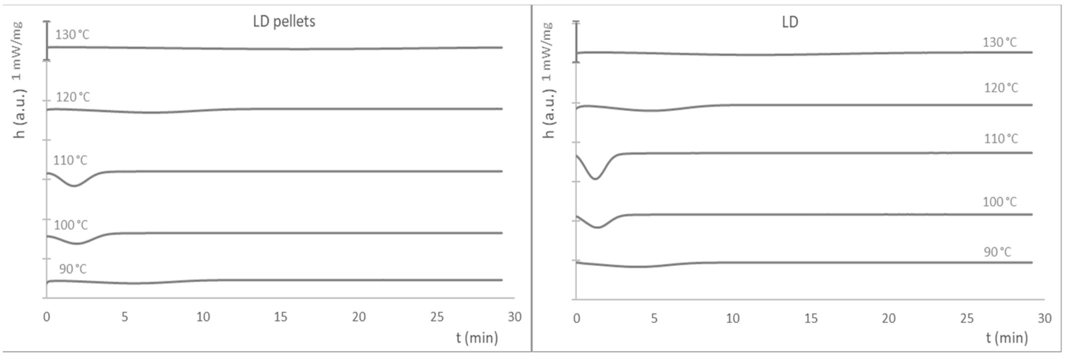

Figure S2. Thermograms obtained for LD materials in the isothermal crystallization assay (left: pellets; right: injection-molded samples)

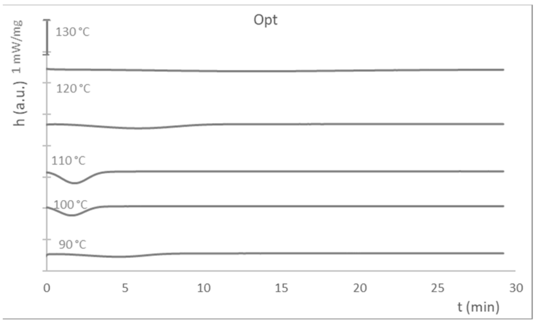

Figure S3. Thermograms obtained for injection molded Opt series in the isothermal crystallization assay

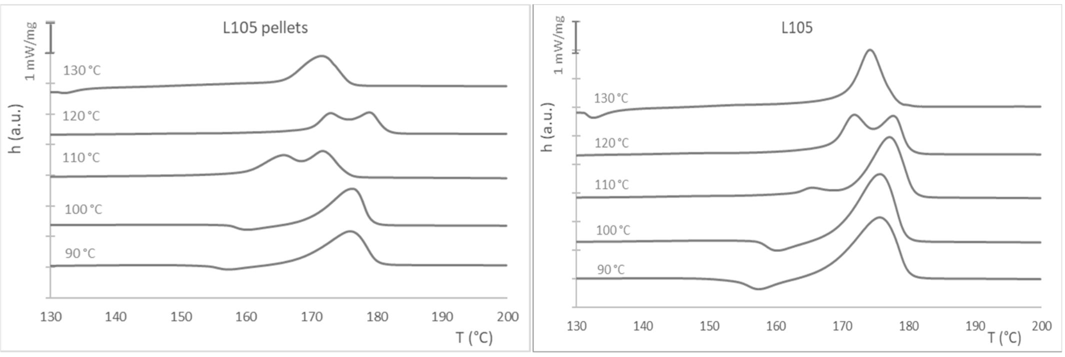

Figure S4. DSC thermograms for L105 materials after the crystallization stage (left: pellets; right: injection-molded samples)

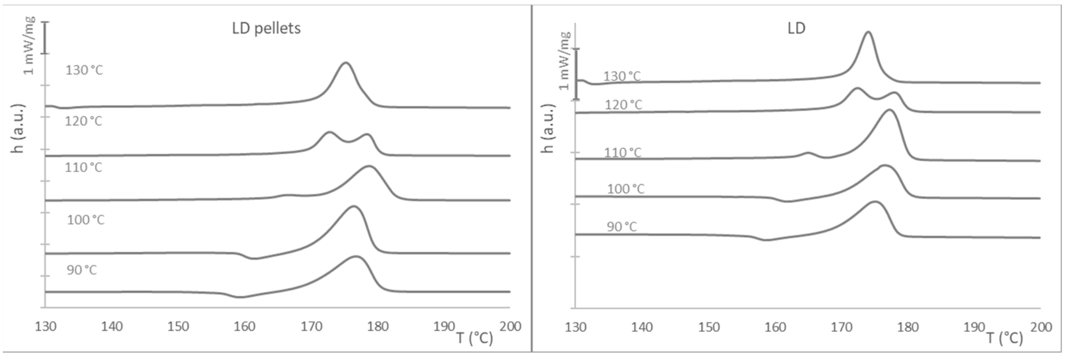

Figure S5. DSC thermograms for LD materials after the crystallization stage (left: pellets; right: injection-molded samples)

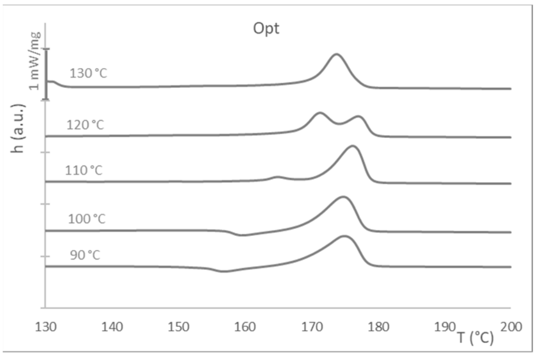

Figure S6. DSC thermograms for Opt materials after the crystallization stage (left: pellets; right: injection-molded samples)

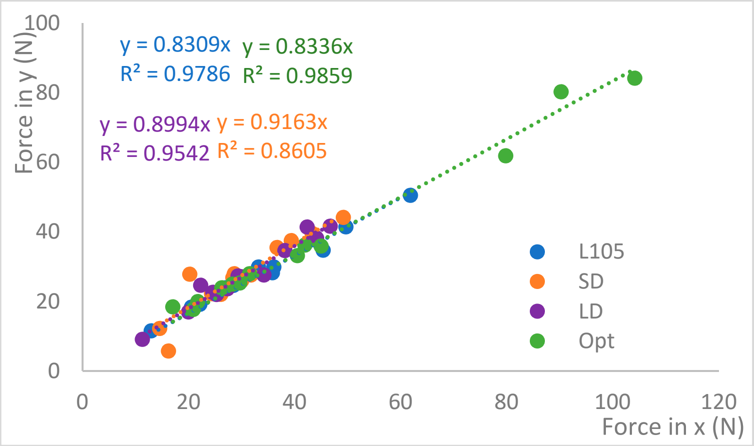

Figure S7. Relationship between force recorded in the x and y axis during stretching

Table S1. Summary of results for the characterization of Opt samples after biaxial stretching (first column: average value; second column: standard deviation)

Short name	Yield strength (MPa)		Tensile modulus (MPa)		Strength at break (MPa)		Thickness (mm)		Strain at yield strength (mm/mm)	
82.2/8.8/56	89.63	1.98	3250.00	50.95	103.66	7.83	0.124	0.016	0.033	0.002
82.2/8.8/79	88.49	2.87	3305.44	132.89	91.13	10.67	0.115	0.014	0.033	0.002
82.2/24.3/79	87.50	2.80	3255.51	180.71	112.14	7.72	0.108	0.017	0.032	0.001
82.2/24.3/56	85.62	6.22	3177.95	161.61	106.76	9.18	0.111	0.020	0.031	0.003
87.5/16.5/68	81.67	5.97	3677.76	152.89	100.69	8.71	0.113	0.021	0.031	0.003
87.5/16.5/90	82.16	2.39	2928.98	150.46	101.34	7.01	0.107	0.006	0.030	0.001
87.5/16.5/45	85.85	5.51	3189.16	213.04	106.64	14.39	0.103	0.019	0.030	0.003
87.5/1.0/68	67.59	3.25	3240.33	177.19	65.04	4.37	0.116	0.027	0.027	0.001
87.5/32.0/68	77.44	3.76	3169.07	173.64	85.99	6.54	0.095	0.015	0.033	0.003
92.8/8.8/56	76.36	3.65	3091.98	323.99	93.62	5.24	0.089	0.022	0.030	0.006
92.8/24.3/56	84.59	2.52	3248.54	261.20	106.95	13.41	0.086	0.012	0.028	0.002

Table S2. Summary of results for the characterization of SD samples after biaxial stretching (first column: average value; second column: standard deviation)

Short name	Yield strength (MPa)		Tensile modulus (MPa)		Strength at break (MPa)		Thickness (mm)		Strain at yield strength (mm/mm)	
82.2/8.8/56	88.47	6.49	3673.08	230.22	96.27	6.43	0.081	0.014	0.032	0.003
82.2/8.8/79	82.77	4.33	2862.05	435.70	80.91	14.62	0.088	0.016	0.032	0.002
82.2/24.3/79	83.18	5.72	3038.31	324.95	84.83	12.23	0.090	0.009	0.031	0.002
82.2/24.3/56	85.36	2.89	3124.51	300.25	97.11	10.11	0.085	0.016	0.031	0.003
87.5/16.5/68	84.41	3.65	3550.15	732.60	90.74	10.02	0.079	0.010	0.032	0.004
87.5/16.5/90	64.55	10.45	2302.21	339.11	63.29	3.93	0.121	0.014	0.027	0.006
87.5/16.5/45	83.78	4.67	3261.35	220.65	104.52	10.84	0.080	0.014	0.028	0.002
87.5/1.0/68	63.90	17.74	3402.51	644.86	63.26	2.51	0.080	0.019	0.030	0.006
87.5/32.0/68	81.08	4.11	3020.90	877.50	89.65	6.82	0.092	0.005	0.033	0.004
92.8/24.3/56	78.53	3.33	2932.390	246.76	71.48	4.39	0.080	0.006	0.029	0.002

Table S3. Summary of results for the characterization of LD samples after biaxial stretching (first column: average value; second column: standard deviation)

Short name	Yield strength (MPa)		Tensile modulus (MPa)		Strength at break (MPa)		Thickness (mm)		Strain at yield strength (mm/mm)	
82.2/8.8/56	85.43	3.29	3494.75	148.09	102.02	12.60	0.083	0.015	0.031	0.002
82.2/8.8/79	86.69	2.25	3715.26	108.76	89.81	15.89	0.084	0.009	0.032	0.003
82.2/24.3/79	87.29	2.56	3642.75	163.76	82.67	8.00	0.087	0.014	0.032	0.003
82.2/24.3/56	87.08	3.17	3703.91	233.05	87.93	8.47	0.084	0.010	0.031	0.002
87.5/16.5/68	77.40	2.88	3263.26	195.88	75.85	6.12	0.101	0.012	0.033	0.003
87.5/16.5/45	87.29	4.65	2733.82	298.54	83.90	13.34	0.073	0.013	0.030	0.002
87.5/1.0/68	64.70	5.80	2668.78	459.29	56.77	2.81	0.089	0.010	0.033	0.005
87.5/32.0/68	86.14	3.75	3264.78	637.97	87.94	7.84	0.084	0.010	0.036	0.005
92.8/24.3/56	76.94	4.64	2253.60	582.69	71.50	8.87	0.077	0.010	0.029	0.002

Table S4. Summary of statistics (p-value and mean difference) for the comparisons of the PLA processed in different conditions and stretched at 82 °C

Comparison		Yield strength		Tensile modulus		Strength at break		Strain at yield strength		Opacity		Force	
		p	Difference	p	Difference	p	Difference	p	Difference	p	Difference	p	Difference
L105 -	Opt	1.000	0.009	0.998	11.251	0.583	-5.095	0.999	0.000	0.002	0.830	0.003	-28.326
	SD	0.073	2.874	1.000	6.859	0.151	8.552	0.594	0.001	< .001	1.894	0.380	11.748
	LD	0.734	1.197	0.126	-162.998	0.225	7.723	0.641	0.001	< .001	2.011	0.388	11.640
Opt -	SD	0.075	2.866	1.000	-4.392	0.005	13.646	0.686	0.001	< .001	1.064	< .001	40.074
	LD	0.738	1.188	0.129	-174.250	0.009	12.818	0.730	0.001	< .001	1.181	< .001	39.966
SD -	LD	0.479	-1.677	0.110	-169.858	0.997	-0.829	1.000	-0.000	0.949	0.117	1.000	-0.108

Table S5. Opacity and maximum forces recorded for PLA injection-molded samples for stretch ratios of 3 and 4

	Opacity (%)				Maximum force in x (N)			
Short name	L105	SD	LD	Opt	L105	SD	LD	Opt
82.2/8.8/56	2.45±0.33	0.43±0.15	0.36±0.14	0.93±0.24	45.443	20.269	22.328	79.871
82.2/8.8/56/4	43.55±0.70	3.55±0.45	3.57±0.58	4.10±0.30	124.514	69.596	64.185	101.756

Table S6. Summary of statistics (p-value and mean difference) for the comparisons of the PLA processed under optimal conditions at stretch ratio (SR) of 3 and 4

Material	Stretch ratio	Yield strength (MPa)		Tensile modulus (MPa)		Strength at break (MPa)		Strain at yield strength (mm/mm)	
		p	Difference	p	Difference	p	Difference	p	Difference
L105	3 vs. 4	0.014	16.711	0.485	-91.841	0.063	4.676	0.160	0.006
SD	3 vs. 4	0.206	8.650	0.027	-414.711	0.188	10.617	0.057	0.006
LD	3 vs. 4	0.469	-3.128	<0.001	-756.084	0.132	17.669	0.530	0.003
Opt	3 vs. 4	0.052	-4.007	<0.001	-785.905	0.102	14.613	0.851	0.000
